# Assessing the integration of refugee health data into national health information systems in Jordan, Lebanon, and Uganda

**DOI:** 10.1186/s13031-024-00608-2

**Published:** 2024-08-05

**Authors:** Lama Bou-Karroum, Najla Daher, Mathilda Jabbour, Laila Akhu-Zaheya, Wejdan Khater, Aladeen Alloubani, Christopher Garimoi Orach, Henry Komakech, Sara Bennett, Fadi El-Jardali

**Affiliations:** 1 Department of Health Management and Policy, Faculty of Health Sciences, American University of Beirut, Beirut, Lebanon; 2 Knowledge to Policy (K2P) Center, American University of Beirut, Beirut, Lebanon; 3 Faculty of Nursing, Jordan University of Science and Technology, Irbid, Jordan; 4 Nursing Research Unit, King Hussein Cancer Center, Amman, Jordan; 5 School of Public Health, College of Health Sciences, Makerere University, Kampala, Uganda; 6 Department of Community Health and Behavioral Sciences, School of Public Health, College of Health Sciences, Makerere University, Kampala, Uganda; 7 Johns Hopkins Bloomberg School of Public Health, Baltimore, MD USA; 8 Department of Health Research Methods, Evidence, and Impact (HEI), McMaster University, Hamilton, ON Canada

**Keywords:** Health information system, Health systems, Refugees, Refugee-sensitive data, Refugee-dense countries, Jordan, Lebanon, Uganda

## Abstract

**Background:**

With the increasing number of protracted refugee crises globally, it is essential to ensure strong national health information systems (HIS) in displacement settings that include refugee-sensitive data and disaggregation by refugee status. This multi-country study aims to assess the degree of integration of refugee health data into national HIS in Jordan, Lebanon, and Uganda and identify the strengths and weaknesses of their national HIS in terms of collecting and reporting on refugee-related health indicators.

**Methods:**

The study employs a comparative country analysis approach using a three-phase framework. The first phase involved reviewing 4120 indicators compiled from global health organizations, followed by a multi-stage refinement process, resulting in 45 indicators distributed across five themes. The second phase consisted of selecting relevant criteria from the literature, including data sources, annual reporting, disaggregation by refugee status, refugee population adjustments, accuracy, and consistency. The third phase involved assessing data availability and quality of the selected indicators against these criteria.

**Results:**

Our analysis uncovered significant challenges in assessing the health status of refugees in Jordan, Lebanon, and Uganda, primarily stemming from limitations in the available health data and indicators. Specifically, we identified significant issues including incomplete local data collection with reliance on international data sources, fragmented data collection from various entities leading to discrepancies, and a lack of distinction between refugees and host populations in most indicators. These limitations hinder accurate comparisons and analyses. In light of these findings, a set of actionable recommendations was proposed to guide policymakers in the three countries to improve the integration of refugee health data into their national HIS ultimately enhancing refugees’ well-being and access to healthcare services.

**Conclusion:**

The current status of refugee-related health data in Jordan, Lebanon, and Uganda indicates the need for improved data collection and reporting practices, disaggregation by refugee status and better integration of refugee health data into national HIS to capture the health status and needs of refugees in host countries. Key improvement strategies include establishing a centralized authority for consistent and efficient data management, fostering transparent and inclusive data governance, and strengthening workforce capacity to manage refugee health data effectively.

**Supplementary Information:**

The online version contains supplementary material available at 10.1186/s13031-024-00608-2.

## Background

Improving national health information systems (HIS) is crucial for providing accurate and up-to-date information for public health decision-making and action [1]. Powerful societal and economic forces are calling for an integrated, patient-centered healthcare information system that allows providers to exchange up-to-date health information quickly and easily and provides timely information to policymakers on the health of the population [2–4]. Such health information systems can reduce healthcare costs, prevent medical errors, improve administrative efficiency, reduce paperwork, and increase access to affordable healthcare [1, 5–8].

With the growing number of protracted refugee crises around the world and given the complexity and variety of modern displacement, ensuring that strong national HIS in displacement settings includes refugee-sensitive data is important to better comprehend and address the health needs of refugees and their barriers to accessing health care [9–14]. As external donors and development agencies have financially contributed to improving health in host countries over the past years, they expect to monitor progress in the programs they support which is impossible without a reliable national HIS that collects comprehensive data on refugees and host populations’ health [1]. Yet, relevant data on refugees’ risk factors, health status, and outcomes are rarely available within the national HIS of refugee-hosting countries [14–18]. This need has been further highlighted recently with the ongoing COVID-19 pandemic, particularly in terms of the inclusion of refugees in preparedness and response plans [14, 15, 18].

Several international organizations have recognized the importance of integrating refugee health data into national HIS to detect systematic disparities in health between different social groups and to measure the impact of policies on health equity and access [11, 14, 19–22]. However, many countries hosting large numbers of refugees still face significant challenges in integrating refugee health data into their national HIS. This is primarily because data collected on refugees in host countries are more focused on infectious diseases and are rarely well integrated into routine health information systems [14, 21, 23–26]. Other challenges include inadequate data collection systems, limited resources for data management and analysis, as well as limited health records being kept for refugees given that the health care they receive is often sporadic and/or incidental [14, 23–26].

Overall, little is known about the capability of national HIS in tracking refugee health data and the extent to which health indicators in refugee-dense countries can be disaggregated between refugee and host populations [11, 25]. This multi-country study aims to fill this gap by examining the refugee-related health indicators in Jordan, Lebanon, and Uganda and identifying the strengths and weaknessesof their national HIS in tracking refugee health.

## Methodology

### Study setting

This multi-country study focuses on three countries: Jordan, Lebanon, and Uganda. These three countries were selected as they all host a large number of refugees and share similarities in terms of the challenges faced at the level of their national health information systems.

### *Lebanon*

Lebanon remains the country hosting the largest number of refugees per capita and per square kilometer in the world since the onset of the Syrian crisis in 2011, with around 169 refugees for every 1000 Lebanese [27, 28]. According to the latest estimates, the Lebanese Government reported that 1.5 million Syrian refugees are residing in Lebanon [29], while the number of refugees registered with the United Nations High Commissioner for Refugees (UNHCR) is 814,715 as of December 2022, reflecting a high number of unregistered Syrian refugees in Lebanon [30].

Prior to the refugee crisis, Lebanon had a fragmented and uncoordinated health information system with multiple data sources scattered across various databases [31], which represented a major challenge for quality checks [32–34]. This situation at the level of the national HIS has been further aggravated by the refugee influx, as the humanitarian system has contributed to further fragmentation in the HIS [34, 35]. While health statistics exist at the national level, they are limited, incomplete, scattered, difficult to access and are not used to inform strategic decision-making [33, 36]. It is evident that data generators are not working together to standardize definitions and baseline values [33]. In reality, government agencies, voluntary organizations, and private researchers are not always keen to share their data and benefit from each other’s experiences [33].

### *Jordan* 

Jordan is one of the countries most hit by the Syrian crisis, with the world's second-greatest proportion of refugees relative to its population [27, 37]. UNHCR reported a total of 661,854 registered Syrian refugees in Jordan as of February 2023 [38]. Jordan has made significant progress in developing its national HIS over the past decade, including the implementation of a national electronic medical record system in public hospitals and health centers [39]. A key initiative in this national effort was the launch of Hakeem, a comprehensive e-health program established in 2009 [39]. This program aimed to revolutionize Jordan's healthcare system by improving healthcare management effectiveness, streamlining workflows, enhancing patient safety standards, and achieving international healthcare standards by the year 2020 [40–42]. However, despite its ambitious goals, the level of adoption and implementation of Hakeem was slower than anticipated [40, 43, 44]. Additionally, the program lacks interoperability with both the UNHCR's health information system and private healthcare facilities at the national level [43, 44]. Challenges also persisted on a broader scale within Jordan's national HIS, including limited financial resources, insufficient regulatory and policy support for health information systems, and a lack of standardized data collection, processing, and analysis methods [45]. There is also a critical shortage of skilled IT professionals equipped to manage and enhance the capabilities of the HIS [46].

### *Uganda*

Since the onset of South Sudan's crisis in December 2013, around 4 million South Sudanese have been displaced, with around 2.26 million fleeing to neighboring countries [47]. Uganda is the world's largest host country for South Sudanese refugees with 865,363 South Sudanese refugees registered with UNHCR as of February 2023 [47]. In recent years, Uganda's Health Management Information System (HMIS) has progressed from a fully paper-based system to an electronic or computer-based system [48, 49]. Furthermore, the Ugandan government has worked tirelessly to improve health record management [49]. This is evidenced by the implementation of "OpenMRS" at the district and sub-district levels, as well as the adoption of District Health Information System 2 (DHIS2) as the National HMIS for collecting health facility data [50]. Notably, the DHIS2 allows for disaggregated reporting by nationality (nationals, refugees, and foreigners) for monthly outpatient department data [51] and has undergone revisions since 2010 to improve performance and accommodate new administrative districts [51, 52]. These advancements have demonstrably improved the timeliness, completeness, and generation of reports that inform clinical decision-making and patient care in Uganda [49]. However, despite these achievements, challenges persist within the country’s health information system [49, 50, 53]. The process of transferring paper-based data from lower-level facilities to the DHIS2 at the district level introduces inaccuracies, delays, and missing information [51]. This is further aggravated by diverse, incompatible, fragmented HIS at various health system levels, as well as a lack of common data standards to promote consistent data exchange [50, 54, 55]. These challenges are compounded by a lack of funding, inadequate ICT facilities, limited training, knowledge gaps, interoperability issues, and user engagement issues [48–50].

### Study approach

The study employs a comparative analysis approach. We sought to assess the availability and quality of key health system indicators over time across the three countries since the onset of the refugee crises. We were interested both in indicators that could describe how the refugee crisis had affected health systems operations (e.g., how the density of health workers or health facilities had changed over time) as well as indicators that revealed differences in health outcomes between host and refugee populations. Across the three countries, we sought to conduct a systematic examination of the strengths and weaknesses of these indicators.

To minimize the impact of confounding factors on our findings, we set the year 2019 as the cutoff point for analysis. This decision was based on two considerations: first, the outbreak of the COVID-19 pandemic in early 2020 which likely affected the national health information system in each of the three countries. Second, the onset of the financial crisis in Lebanon towards the end of 2019, also had a negative impact on the national health information system regardless of the refugee crises.

### Framework for comparative analysis

A structured framework was developed to systematically assess the national HIS of Jordan, Lebanon, and Uganda regarding their integration and management of refugee health data. This framework is comprised of three distinct phases: the selection of a comprehensive set of indicators, the selection of criteria for assessment, and the assessment of each indicator against the criteria.

### *Selection of indicators*

The first phase involved selecting relevant health system indicators that would provide a foundation for assessing the integration and efficacy of handling refugee health data in the three countries. We conducted an extensive review of indicators lists from reputable organizations, including the World Health Organization (WHO), Centers for Disease Prevention and Control (CDC), United Nations Statistics Division, The Pan American Health Organization (PAHO), The Organization for Economic Co-operation and Development (OECD); and The World Bank (WB) [36, 56–64]. This comprehensive search resulted in a total of 4,120 health-related indicators.

**Fig. 1:**
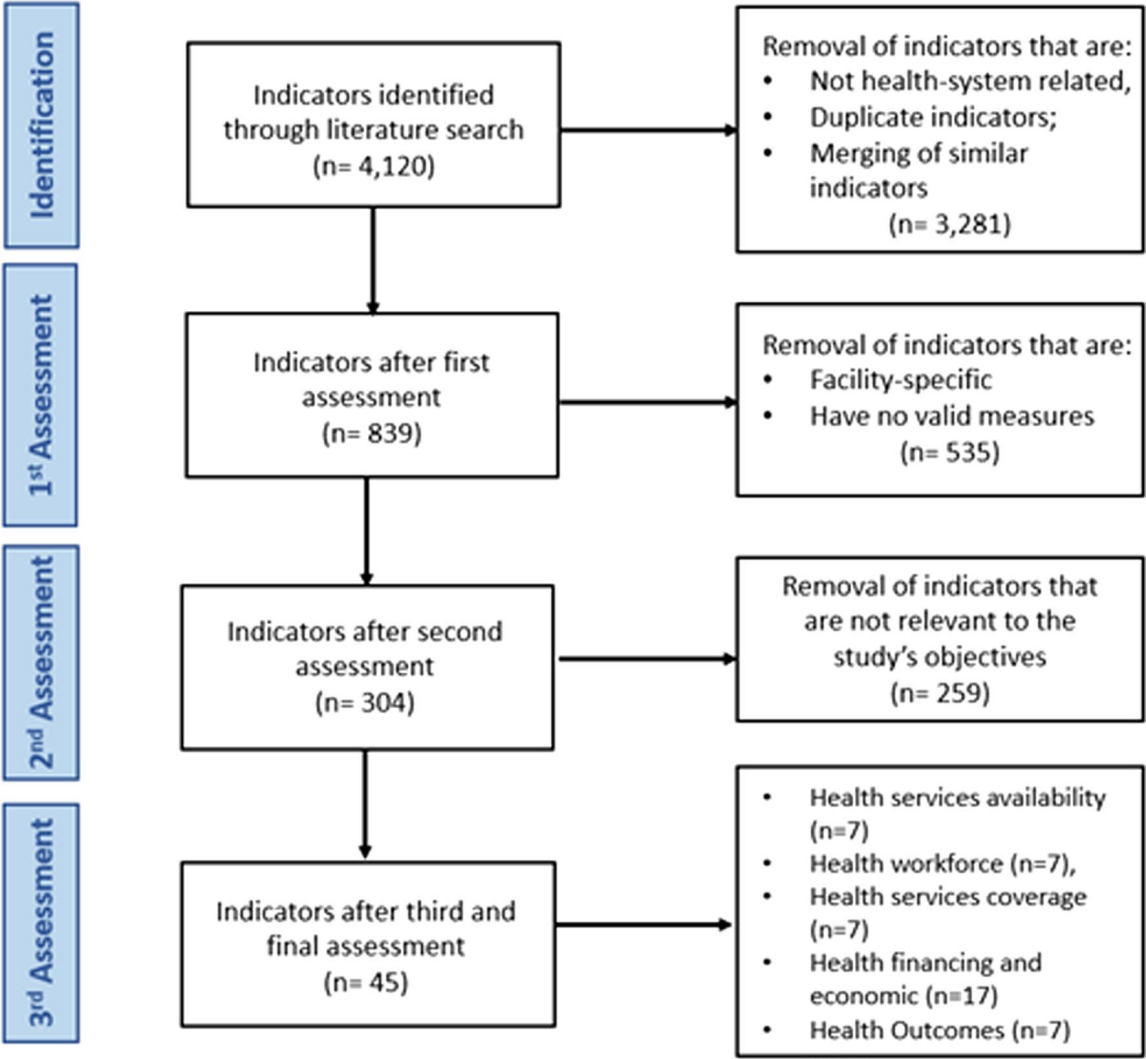
Flowchart detailing the process of indicators selection

A multi-stage refinement process was then employed to determine a focused set of indicators most relevant to the study objectives. A first assessment was conducted to exclude indicators that were not health system-related, remove duplicate indicators, and merge similar indicators; this resulted in a total of 839 indicators. A second assessment was conducted to remove facility-specific indicators and indicators with no valid measures; this resulted in a total of 304 indicators. Figure 1 details the selection process of these indicators.

To ensure the final set of indicators possessed the necessary level of relevance and comprehensiveness, experts in health systems and refugee health were consulted. These experts conducted a meticulous assessment of each remaining indicator, focusing on its relevance in understanding disparities in access to and utilization of healthcare services between host and refugee populations. Following this rigorous selection process, a total of 45 indicators were included for analysis and categorized under five themes representing the main building blocks of health systems: Health services availability (n = 7), Health workforce (n = 7), Health services coverage (n = 7), Health financing (n = 17), and Health outcomes (n = 7) (See Appendix I for the list of all included indicators, their definitions and their identified strengths and weaknesses across the three countries).

### *Selection of assessment criteria*

To effectively assess and compare the chosen health system indicators across Jordan, Lebanon, and Uganda, a set of assessment criteria was adopted from the literature [65, 66] and employed as follows:

- **Data sources:** This criterion examines the accessibility of data and the degree to which it is publicly available. It considers the availability of health data from local entities such as government bodies, statistics departments, Ministries of Health, national Health Management Information Systems (HMIS), and national health surveys. Additionally, it examines the availability of data from international entities such as the WHO, UNHCR, and the World Bank.
- **Annual reporting:** This criterion focuses on the frequency of data collection and reporting. It assesses whether data is collected and reported on an annual basis, both prior to and after the start of the refugee crisis. Regular reporting ensures the availability of up-to-date information for monitoring and evaluating the health system's performance.
- **Disaggregation by refugee status:** This criterion emphasizes the importance of collecting and reporting data that is disaggregated by refugee status, among other relevant factors. It ensures that data distinguishes between the refugee population and the host population, allowing for a specific analysis of the health situation and needs of refugees.
- **Refugee Population Adjustment:** This criterion emphasizes the adjustment made in the calculation to account for the presence and impact of the refugee population on the health system indicators. This criterion examines whether the reported data includes the refugee population in both the numerator and denominator of the indicators being calculated or not.
- **Accuracy:** Accuracy refers to the degree to which the collected data is free from errors or biases. It focuses on the absence of outliers and ensures that the data accurately reflects the health system's performance over time. Robust data validation processes and quality assurance measures contribute to maintaining data accuracy.
- **Consistency:** Consistency examines the standardization and comparability of data across different sources and time periods. It evaluates the presence of discrepancies or variations when the same data is available from both local and international sources. Consistency in data allows for reliable comparisons and analysis of health system indicators.

### *Data collection and analysis*

This section details the data collection and analysis processes employed in the third phase of the framework. The primary objective was to gather information on the pre-selected indicators for the three countries, followed by an assessment of their quality against the established criteria.

To ensure consistent and accurate data collection, a standardized data extraction form was developed. This form facilitated the extraction of relevant information and data points for each of the 45 indicators across the three countries for the period 2008–2019 (See Appendix II for the data collection form). Microsoft Excel served as a supplementary tool for basic data analysis. Line and bar graphs were also generated to visually assess trends and facilitate comparisons in refugee data integration practices across the three countries.

Two researchers, N.D. and M.J., independently utilized the standardized form to extract data and assess each indicator against the defined six criteria (detailed below). Then, they collaboratively discussed any discrepancies arising from the assessment process to arrive at a consensus. In cases where disagreements persisted beyond initial discussion, a third senior researcher, L.B.K., acted as a final arbiter.

- **Data Sources (Criterion 1):** The first criterion evaluated the availability of data for each indicator across the three countries. To achieve this, a comprehensive search was conducted across both local and international sources. Local sources encompassed official websites of the respective Ministries of Health, national vital registration systems, routine health service data, health surveys, and population-based surveys. Additionally, official reports, annual statistical reports, and national health accounts were reviewed for relevant information. International sources included databases from the WHO Global Health Observatory, the World Bank, UNHCR, and UNICEF among others. Additional targeted searches were conducted using Google Scholar and the general Google search engine. Following this extensive search, each indicator was categorized based on the data source(s) where it was found: local sources only, international sources only, both local and international sources, or not reported in any identified sources. If no data was obtainable from any source for a specific indicator, the remaining five criteria could not be assessed, and such indicators were marked "not applicable”.
- **Annual Reporting (Criterion 2):** To assess the frequency of reporting for each indicator, data points were extracted for each year between 2008 and 2019. Indicators with consistent and complete reporting throughout the entire period were categorized as "yes". Conversely, those with any interruptions or missing data points within the 12-year timeframe were marked as "no”.
- **Disaggregation by Refugee Status (Criterion 3):** This criterion evaluated whether the reported data distinguished between the refugee population and the host population. Indicators reported for both populations separately were categorized as "yes," since this level of disaggregation allows for targeted analysis of refugee health needs and identification of potential disparities in access to healthcare services. Indicators that did not disaggregate data by refugee status were categorized as "no."
- **Refugee Population Adjustment (Criterion 4):** This assessment focused on whether the reported data for each indicator included the refugee population in both the numerator and denominator used for calculations. Due to the potentially complex nature of this criterion, validation was conducted in collaboration with experts and representatives from statistics departments responsible for data collection and reporting in each of the three countries. These individuals were chosen for their extensive knowledge and experience within the national HIS of the host countries. Their expertise ensured a clear understanding of data reporting practices and facilitated the accurate categorization of indicators based on this criterion.
- **Accuracy of Reported Data (Criterion 5):** The accuracy of reported data was evaluated by visually inspecting line and bar graphs for each indicator. The presence of unexplained outliers, such as sudden increases or decreases in a particular year, indicated potential inaccuracies and led to a "no" categorization for that specific indicator.
- **Consistency of Reported Data (Criterion 6):** This criterion assessed the consistency of data reported from multiple sources. When data for an indicator was available from more than one source (local or international), the data points were examined for standardization and comparability across different sources and time periods. Any observed discrepancies or variations resulted in a categorization of "no" for consistency.

A final validation process was conducted to enhance the robustness of the data assessment further. This process involved collaborating with the experts and representatives from statistics departments from the respective countries. Their local knowledge and expertise provided valuable insights and ensured the accuracy of the categorization for each indicator based on the defined criteria.

## Results

### Health services availability indicators (n = 7)

**Fig. 2:**
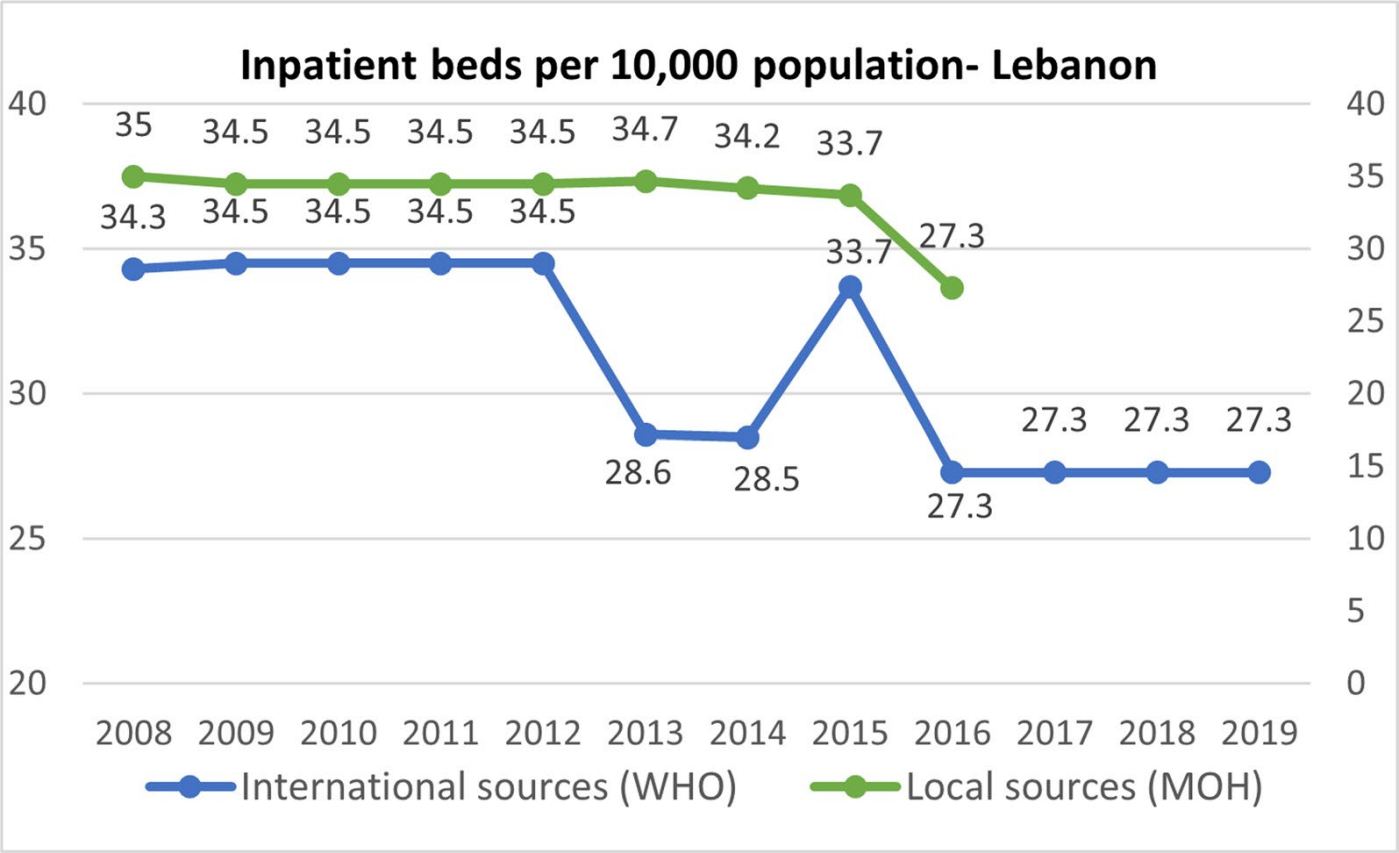
Total hospital beds per 10,000 population in Lebanon (2008–2019) [67, 68]. * Last published data in 2016 from local sources

**Fig. 3:**
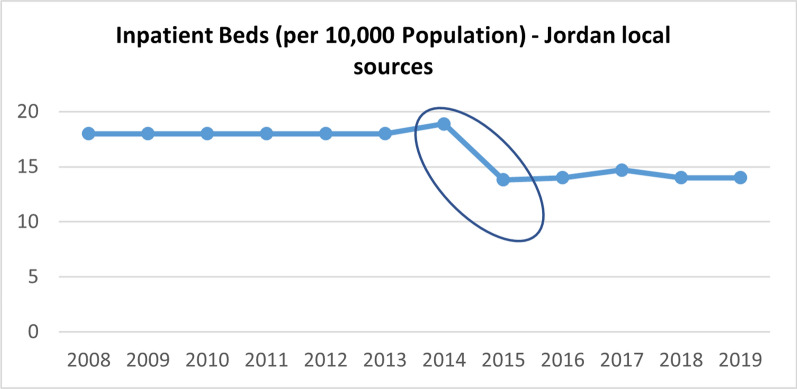
Total hospital beds per 10,000 population in Jordan (2008–2019) [69]

Table 1 presents a comprehensive assessment of the strengths and weaknesses of health service availability indicators in Jordan, Lebanon, and Uganda. Our analysis reveals that none of the health service availability indicators across the three countries are disaggregated by refugee status. This lack of disaggregation poses a significant challenge to analyzing the specific health services available to refugees in comparison to the host population. Furthermore, none of the indicators under this theme account for the refugee population in these countries, except for the number of inpatient beds per 10,000 population in Jordan and Lebanon, which started accounting for the Syrian refugee population in 2015 and 2016 respectively. This change in methodology for calculation explains the sudden drop in density observed for the two countries, as depicted in Figs. 2 and 3 below. It is important to note that this indicator was last reported in 2016 from local sources in Lebanon, limiting the availability of data for proper comparison with only one year accounting for the refugee population.

**Table 1:** Strengths and weaknesses of health services availability indicators in Jordan, Lebanon and Uganda (n = 7)

Health services availability	Data sources			Annual reporting			Disaggregation by refugee status			Refugee population adjustment			Accuracy			Consistency		
	JOD	LBN	UG	JOD	LBN	UG	JOD	LBN	UG	JOD	LBN	UG	JOD	LBN	UG	JOD	LBN	UG
Number and distribution of health facilities per 10,000 population	✕	✕	✕	–	–	–	–	–	–	–	–	–	–	–	–	–	–	–
Number of inpatient beds per 10,000 population	I & L	I & L	I & L	*✓*	✓^*^	*✓*	✕	✕	✕	*✓*^b^	*✓*^b^	✕	*✓*	✕	*✓*	*✓*	✕	✕
Number and Percentage of health facilities supported by humanitarian organizations	✕	✕	✕	–	–	–	–	–	–	–	–	–	–	–	–	–	–	–
Density of district/rural hospitals (per 100,000 population)	I	I	I & L	✕	✕	*✓*	✕	✕	✕	✕	✕	✕	✕	✕	✕	–	–	✕
Density of primary health care facilities	I & L^a^	I & L^a^	L	*✓* ^a^	*✓* ^a^	*✓*	✕	✕	✕	✕	✕	✕	–	–	✕	–	–	–
Maternity bed density per 1000 pregnant women	✕	✕	✕	–	–	–	–	–	–	–	–	–	–	–	–	–	–	–
Number of outpatient department visits per 10,000 population per year	✕	✕	L	–	–	✕	–	–	✕	–	–	✕	–	–	✕	✕	✕	–

JOD, Jordan; LBN, Lebanon; UG, Uganda

*✓*, Yes; ✕, No/Not available; –, Not Applicable/Cannot be assessed

I: International source only; L: Local source only

I & L: Both international and local sources available

*Last published data in 2016

^a^The indicator is reported as number only and not as density from local sources

^b^Started accounting for refugee population in 2015 for Jordan and in 2016 for Lebanon

In terms of data availability from local sources, only one indicator is reported annually across the three countries, namely the number of inpatient beds per 10,000 population. However, caution must be exercised when interpreting this indicator due to the presence of several outliers. In Jordan, outliers were observed around the year 2015, coinciding with the latest population census conducted in November 2015, where total population calculations started accounting for the Syrian refugee population, four years after the onset of the Syrian refugee crisis (Fig. 3). Similarly, outliers were observed between the years 2012 and 2016 in Lebanon, for both local and international sources. One possible explanation for these outliers, in addition to the adjustment made to account for Syrian refugees' populations starting in 2015 (Fig. 2), could be errors in data collection, data entry, or data reporting.

It is important to note that this indicator is also reported by international sources for the three countries. However, discrepancies were observed between the values reported by different sources for the same years, particularly for Lebanon (Fig. 2) and Uganda. These inconsistencies pose challenges to ensuring reliable comparisons and analysis of this health system indicator. On the other hand, it is important to highlight that for Jordan, there were no discrepancies between the reported values from local sources and international sources (Ministry of Health, World Health Organization, and World Bank) thus data under this indicator were consistent across the different sources.

In addition to inpatient bed density, other indicators reported from local sources in at least one of the three countries include the density of primary healthcare facilities, the density of district/rural hospitals per 100,000 population, and the number of outpatient department visits per 10,000 population, which were reported from local sources only for Uganda, as shown in Table 1. However, it is important to note that for both Lebanon and Jordan, the primary healthcare facilities indicator is reported as a number rather than as density from local sources. Consequently, this number-based indicator does not provide a comprehensive assessment of primary care facility availability relative to the total population. Since international sources report this indicator as density, a comparison between the two values cannot be made, thus hindering the assessment of accuracy and consistency.

On the other hand, only one indicator, the density of district/rural hospitals (per 100,000 population), was reported exclusively by international sources, specifically by the World Health Organization's Global Health Observatory (GHO), for Lebanon and Jordan. Several shortcomings were identified regarding this indicator, including the lack of annual reporting, the absence of disaggregation by refugee status, and the failure to account for refugees in total population calculations. These limitations primarily stem from the reliance on international sources for estimates and extrapolations, which do not adequately consider the refugee populations.

It is worth noting that three indicators under health service availability were not available from either local or international sources. These indicators include the number and distribution of health facilities per 10,000 population, the number and percentage of health facilities supported by humanitarian organizations, and the maternity bed density per 1000 pregnant women. Furthermore, the number of outpatient department visits per 10,000 population per year was not reported for both Jordan and Lebanon, further hindering our ability to comprehensively assess outpatient care accessibility in these countries. In Uganda, this indicator was available from local sources; however, it was only reported for two years (2013 and 2014).

### Health workforce indicators (n = 7)

Table 2 presents the strengths and weaknesses of health workforce indicators in Jordan, Lebanon, and Uganda. Two indicators were unavailable from either local or international sources in all three countries. Specifically, the number of health workers per 10,000 population and the number of medical and pathology laboratory technicians were not available from any source in Jordan and Lebanon, thus hindering their assessment. While the number of medical and pathology laboratory technicians was available from international sources for Uganda (GHO), it was only reported for two years (2015 and 2018), not disaggregated by refugee status, and did not account for the refugee population.

**Table 2:** Strengths and weaknesses of health workforce indicators in Jordan, Lebanon and Uganda (n = 7)

Health workforce	Data sources			Annual reporting			Disaggregation by refugee status			Refugee population adjustment			Accuracy			Consistency		
	JOD	LBN	UG	JOD	LBN	UG	JOD	LBN	UG	JOD	LBN	UG	JOD	LBN	UG	JOD	LBN	UG
Number of health workers per 10,000 population [SDG 3.c.1]	✕	✕	✕	–	–	–	–	–	–	–	–	–	–	–	–	–	–	–
Density of Physicians (per 10,000 population)	I & L	I & L	I & L	*✓*	*✓*	*✓*	✕	✕	✕	*✓*^a^	✕	✕	*✓*	*✓*	✕	✕	✕	✕
Density of Dentists (per 10,000)	I & L	I & L	I	*✓*	*✓*	✕	✕	✕	✕	*✓*^a^	✕	✕	✕	✕	–	✕	✕	–
Density of nursing and midwifery personnel (per 1000 population)	I & L	I & L	I & L	*✓*	*✓*	*✓*	✕	✕	✕	*✓*^a^	✕	✕	*✓*	*✓*	*✓*	✕	✕	*✓*
Density of Pharmacists (per 10,000)	I & L	I & L	I	*✓*	*✓*	✕	✕	✕	✕	*✓*^a^	✕	✕	✕	✕	–	✕	✕	–
Number of Medical and Pathology Laboratory Technicians	✕	✕	I	–	–	✕	–	–	✕	–	–	✕	–	–	–	–	–	–
Psychiatrists working in mental health sector (p*✓*er 100,000)	I	I	I	✕	✕	✕	✕	✕	✕	✕	✕	✕	–	–	–	–	–	–

JOD, Jordan; LBN, Lebanon; UG, Uganda

*✓*, Yes; ✕, No/Not available; –, Not Applicable/Cannot be assessed

I, International source only; L, Local source only

I & L, Both international and local sources available

^a^Started accounting for refugee population in 2015

**Fig. 4:**
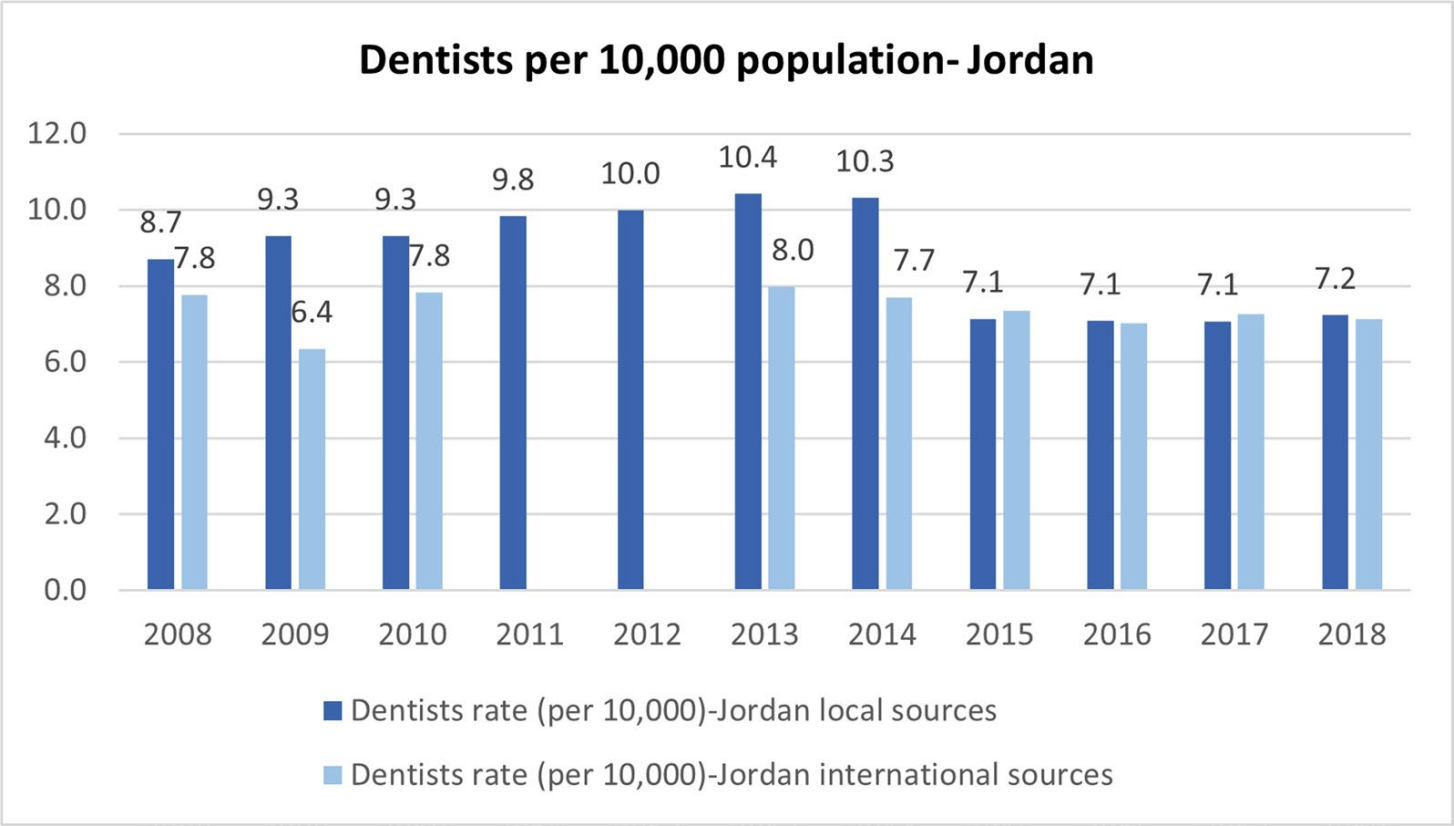
Dentists rate per 10,000 population in Jordan (2008–2018) [69, 70]

Out of the seven indicators under the health workforce building blocks, only two were available from both local and international sources across all three countries. In Jordan, the local data accounted for the refugee population starting from 2015. However, discrepancies between the local and international sources in Jordan raised concerns about consistency, and the data was not segregated by refugee status. Notably, an outlier was observed for the health workforce indicators reported in Jordan for the year 2015, reflecting a change in calculation coinciding with the latest population census conducted in 2015, which began accounting for the Syrian refugee population. Figure 4 below provides an example from Jordan.

**Fig. 5:**
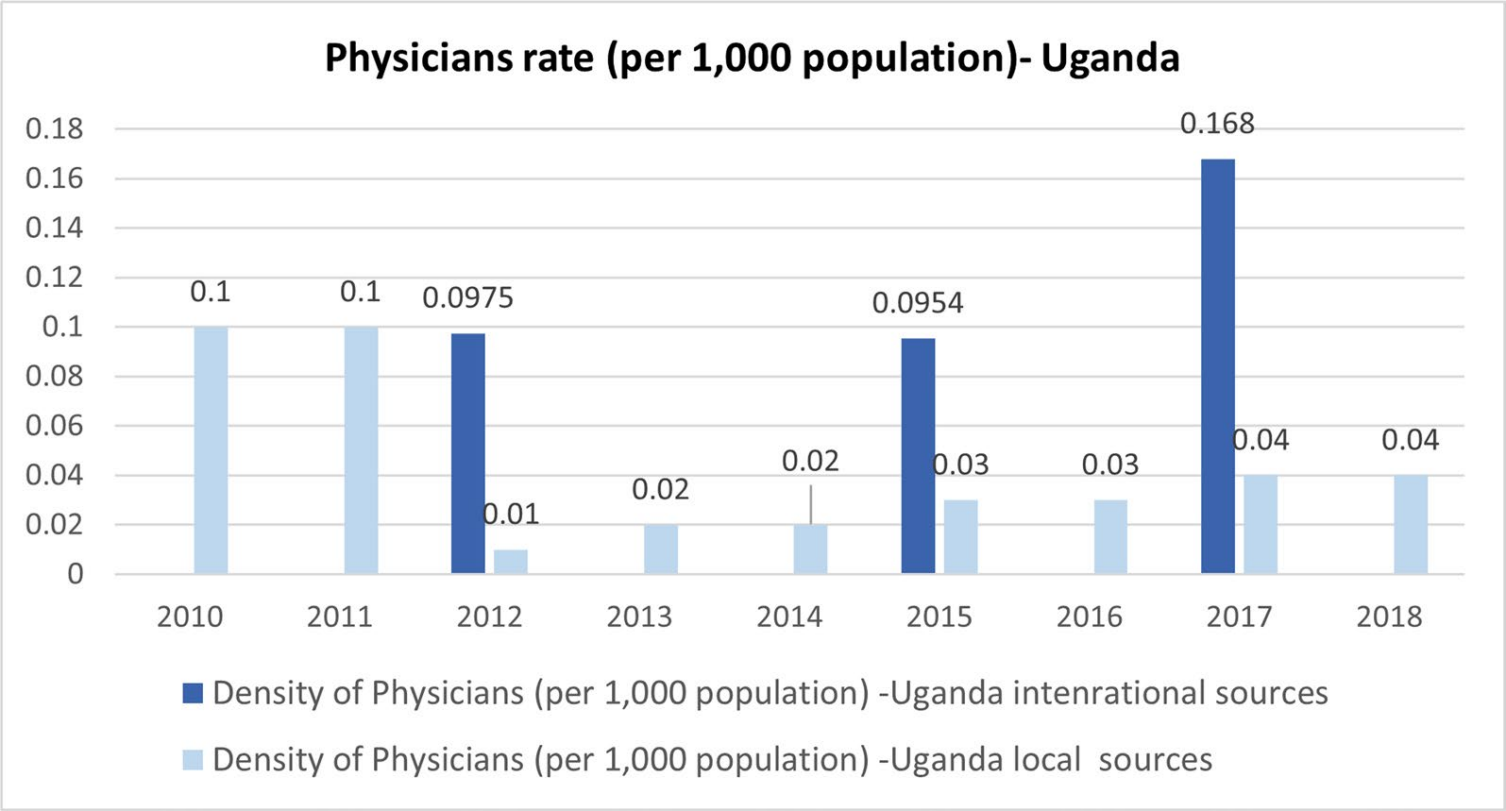
Density of physicians per 10,000 population in Uganda (2010–2019)[71, 72]

In terms of consistency, significant disparities were evident between locally reported data and data from international sources for all three countries when data was available from different sources, as depicted in Figs. 4 and 5.

Two other indicators, the Density of Dentists (per 10,000) and the Density of Pharmacists (per 10,000), were obtainable from both local and international sources in Jordan and Lebanon. However, for Uganda, these indicators were solely available from international sources and were not reported annually.

Limited information exists regarding mental health professionals in the three countries, and data could only be found in international reports and studies. Specifically, the number of psychiatrists working in the mental health sector (per 100,000), was accessible solely from international sources for all three countries. Also, this indicator was not reported annually, was not segregated by refugee status, and did not account for the refugee population across the three countries.

### Health services coverage and access indicators (n = 7)

Table 3 presents an overview of the strengths and weaknesses of health services coverage and access indicators in Jordan, Lebanon, and Uganda. Across the three countries, all seven indicators under the health services coverage and access were available either from local or international sources, with the exception of the primary health care utilization threshold which was not available in Uganda, and the antenatal care coverage which was not available in Lebanon. The contraceptive prevalence rate was only reported from international sources across the three countries and lacked annual reporting, disaggregation by refugee status, and inclusion of the refugee population in the calculation. Furthermore, two indicators, namely births attended by skilled health personnel and access to a core set of relevant essential medicines, were solely sourced from international data in Jordan and Lebanon. In the case of births attended by skilled health personnel in Lebanon, the data was disaggregated between Lebanese and non-Lebanese populations; however, it was only available for a single year (2018), limiting meaningful comparison and analysis.

**Table 3:** Strengths and weaknesses of health services coverage and access indicators in Jordan, Lebanon and Uganda (n = 7)

Health services coverage and access	Data Sources			Annual reporting			Disaggregation by Refugee status			Refugee population adjustment			Accuracy			Consistency		
	JOD	LBN	UG	JOD	LBN	UG	JOD	LBN	UG	JOD	LBN	UG	JOD	LBN	UG	JOD	LBN	UG
Primary health care utilization threshold	L	L	✕	*✓*^α^	*✓* ^α^	–	✕	✕	–	*✓* ^α^	*✓* ^α^	–	–	–	–	–	–	–
Coverage of DTP3	I & L	I & L	I & L	*✓*	*✓*	*✓*	✕	✕	✕	*✓*	*✓*	*✓*	✕	*✓*	✕	*✓*	*✓*	*✓*
Contraceptive prevalence rate	I	I	I	✕	✕	✕	✕	✕	✕	✕	✕	✕	–	–	–	–	–	–
Antenatal care coverage	I	✕	L	✕	✕	*✓*	✕	–	✕	✕	–	*✓*	✕	–	✕	–	–	–
Percentage of deliveries by caesarean section	I	I & L	I & L	✓^*^	*✓*	*✓*	✕	✕	✕	✕	✕	✕	*✓*	*✓*	✕	–	*✓*	✕
Births attended by skilled health personnel [SDG 3.1.2]	I	I	I & L	✕	✕	✕	✕	*✓*^a^	✕	✕	✕	*✓*	–	*✓*	✕	–	–	✕
Access to a core set of relevant essential medicines [SDG 3.b.3]	I	I	L	✕	✕	*✓*	✕	✕	✕	✕	✕	✕	–	–	✕	–	–	–

JOD, Jordan; LBN, Lebanon; UG, Uganda

*✓*, Yes; ✕, No/Not available; – Not Applicable/Cannot be assessed

I, International source only; L, Local source only

I & L, Both international and local sources available

^*^ No data published after the year 2017

^a^Indicator is disaggregated by Lebanese and non-Lebanese population for one year only (2018)

**Fig. 6:**
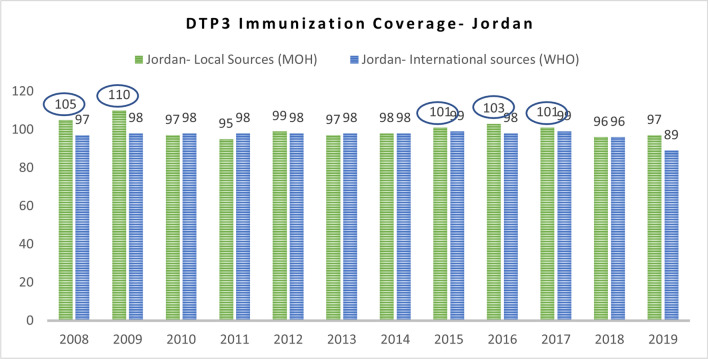
DTP3 immunization coverage in Jordan (2008–2019) [69, 73]

Discrepancies between locally reported data and data from international sources were observed when both sources were available, primarily due to variations in data collection methods. Among the indicators, the coverage of the DTP3 vaccine was the only one reported annually from local sources, with comprehensive data across all three countries. However, the quality of data for this indicator in Jordan raised concerns, as the reported values exceeded 100% for several years, including 2008, 2009, 2015, 2016, and 2017, as illustrated in Fig. 6 below. Another shortcoming of this indicator across the three countries is that the reported data is not disaggregated by refugee status although the reported values regarding the coverage of the DTP3 vaccine account for the refugee populations in the three countries.

### Health financing and economic indicators (n = 17)

Table 4 presents the strengths and weaknesses of health financing and economic indicators in Jordan, Lebanon, and Uganda. In Lebanon, the National Health Accounts were not published for the years 2011, 2013, and 2014, and the most recent available data is from 2017. Similarly, for Jordan, the last published national health account is from 2017, and there are missing data reports for certain indicators in 2014. This lack of updated data significantly limits the assessment of health financing indicators for both Jordan and Lebanon. On the other hand, Uganda has more updated data on health financing and economic indicators from local sources, as the Ministry of Health recently published one report covering three fiscal years (2016/17, 2017/18, and 2018/19).

**Table 4:** Strengths and Weaknesses of health financing and economic indicators in Jordan, Lebanon and Uganda (n = 17)

Health financing and economic indicators	Data Sources			Annual reporting			Disaggregation by Refugee status			Refugee population adjustment			Accuracy			Consistency		
	JOD	LBN	UG	JOD	LBN	UG	JOD	LBN	UG	JOD	LBN	UG	JOD	LBN	UG	JOD	LBN	UG
Current Health Expenditure (CHE) as % Gross domestic product (GDP)	I & L	I & L	I & L	✓^*^	✓^*^	✓^*^	✕	✕	✕	✕	✕	✕	*✓*	*✓*	*✓*	✕	✕	✕
Current Health Expenditure (CHE) per Capita in US$	I & L	I & L	I & L	✓^*^	✓^*^	✓^*^	✕	✕	✕	✕	✕	✕	*✓*	*✓*	*✓*	✕	✕	✕
Domestic Health Expenditure as % of Current Health Expenditure	✕	✕	✕	–	–	–	–	–	–	–	–	–	–	–	–	–	–	–
Domestic General Government Health Expenditure as % Current Health Expenditure	I & L	I & L	I & L	✓^*^	✓^*^	✓^*^	✕	✕	✕	✕	✕	✕	*✓*	*✓*	*✓*	✕	✕	✕
Domestic General Government Health Expenditure per Capita in US$	I	I	I	*✓*	*✓*	*✓*	✕	✕	✕	✕	✕	✕	✕	✕	✕	–	–	–
Domestic Private Health Expenditure as % Current Health Expenditure	I & L	I & L	I & L	✓^*^	✓^*^	✓^*^	✕	✕	✕	✕	✕	✕	*✓*	*✓*	*✓*	✕	✕	✕
Domestic Private Health Expenditure per Capita in US$	I	I	I	*✓*	*✓*	*✓*	✕	✕	✕	✕	✕	✕	*✓*	*✓*	*✓*	–	–	–
Health Expenditure from External Sources as % of Current Health Expenditure	I & L	I & L	I & L	✓^*^	✓^*^	✓^*^	✕	✕	✕	✕	✕	✕	*✓*	*✓*	*✓*	✕	✕	✕
Health Expenditure from External Sources per Capita in US$	✕	✕	✕	–	–	–	–	–	–	–	–	–	–	–	–	–	–	–
Out of pocket Expenditures as % Current Health Expenditure	I & L	I & L	I & L	✓^*^	✓^*^	✓^*^	✕	✕	✕	✕	✕	✕	*✓*	*✓*	*✓*	✕	✕	✕
Out-of-Pocket Expenditure per Capita in US$	✕	✕	✕	–	–	–	–	–	–	–	–	–	–	–	–	–	–	–
Gross Domestic Product per Capita in US$	I & L	I & L	I & L	✓^*^	✓^*^	✓^*^	✕	✕	✕	✕	✕	✕	*✓*	*✓*	*✓*	✕	✕	✕
Primary Health Care (PHC) Expenditure per Capita in US$	✕	✕	✕	–	–	–	–	–	–	–	–	–	–	–	–	–	–	–
Primary Health Care (PHC) Expenditure as % Current Health Expenditure (CHE)	✕	✕	✕	–	–	–	–	–	–	–	–	–	–	–	–	–	–	–
Domestic General Government Expenditure on primary health care as % Domestic General Government Health Expenditure	✕	✕	✕	–	–	–	–	–	–	–	–	–	–	–	–	–	–	–
Proportion of the population with impoverishing health expenditure	✕	✕	✕	–	–	–	–	–	–	–	–	–	–	–	–	–	–	–
Proportion of the population with large household expenditure on health as a share of total household consumption or income [SDG 3.8.2]	✕	✕	✕	–	–	–	–	–	–	–	–	–	–	–	–	–	–	–

JOD, Jordan; LBN, Lebanon; UG, Uganda

*✓*, Yes; ✕, No/Not available; –, Not Applicable/Cannot be assessed

I, International source only; L,: Local source only

I & L, Both international and local sources available

*Reported annually from international sources but not reported annually from local sources/missing some years from local sources

Regularly conducting National Health Accounts is crucial as it provides a systematic, consistent, and comprehensive methodology for monitoring financial flows within the health sector. This tool was specifically developed to inform the health policy process. The availability of financing information assists in the preparation of health sector strategic plans, resource mobilization, and the formulation of appropriate health financing policies, enabling efficient resource allocation.

Regarding refugee-specific health financing and economic indicators, no local data were found across the three countries. Such data can only be obtained from international reports, such as the Vulnerability Assessment of Syrian Refugees in Lebanon, jointly published by UNICEF, UNHCR, and WFP. However, relying solely on these reports and studies has limitations, as the samples used may not be representative of the entire refugee population residing in the host country. Especially since the characteristics of unregistered refugees differ from those registered with UNHCR (which are the only ones represented in such reports), further affecting the generalizability of the findings.

Discrepancies were observed between locally and internationally reported data when both sources were available for the three countries. Specifically, discrepancies were observed for seven indicators, including the current health expenditure (CHE) as % gross domestic product (GDP), current health expenditure (CHE) per capita in US$, domestic general government health expenditure as % current health expenditure, domestic private health expenditure as % current health expenditure, health expenditure from external sources as % of current health expenditure, out of pocket expenditures as % current health expenditure and gross domestic product per capita in US$.

### Health outcomes indicators (n = 7)

**Fig. 7:**
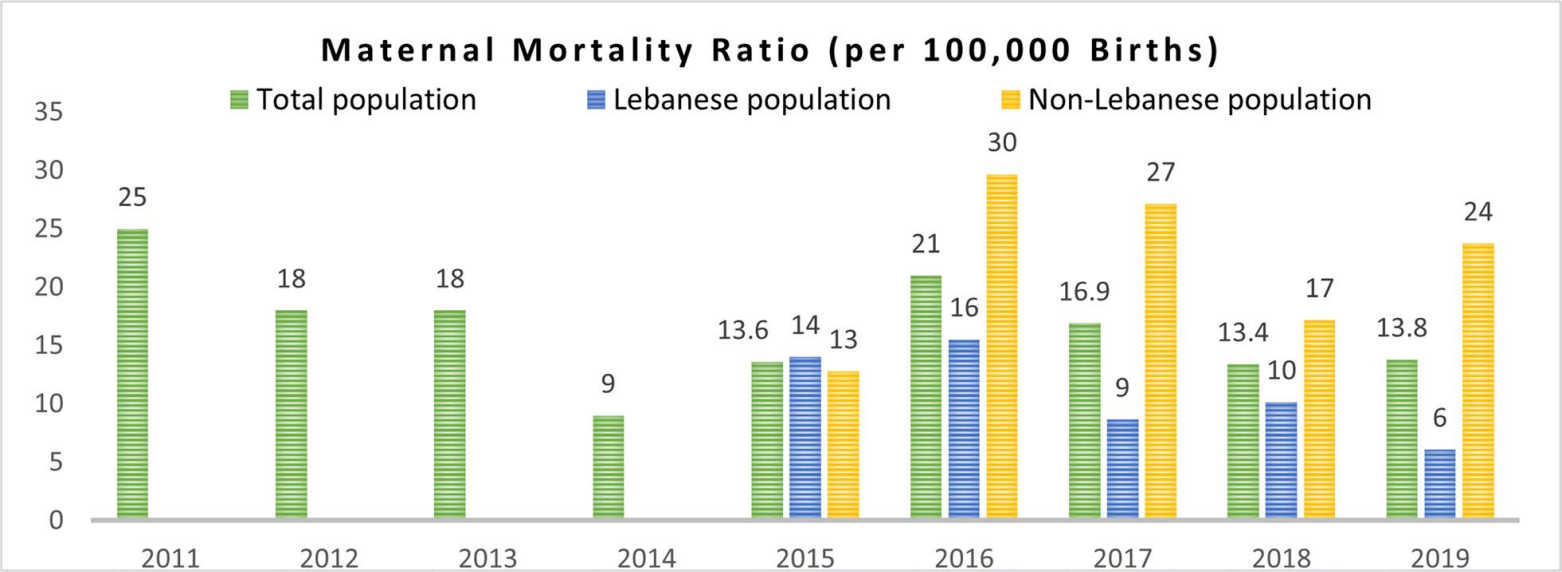
Maternal mortality rate per 100,000 live births in Lebanon (2008–2019)[67]

Table 5 presents the strengths and weaknesses of health outcomes indicators in Jordan, Lebanon, and Uganda. It is noteworthy that all seven indicators under this theme were available from either local or international sources across the three countries. Two indicators, infant mortality rate and maternal mortality ratio, were reported annually from both local and international sources for all three countries. In Jordan, the local data for these indicators started accounting for the Syrian refugee population in 2015, while in Lebanon, the data was disaggregated by nationality (Lebanese and non-Lebanese), with the non-Lebanese category including the Syrian refugees, as depicted in Fig. 7. Although neonatal and maternal mortality data are stratified by nationality in Lebanon (reported for Lebanese and for non-Lebanese) such stratification was only available for the years 2015–2018 and with no additional stratification by nationality for the non-Lebanese data.

**Table 5:** Strengths and weaknesses of health outcomes indicators in Jordan, Lebanon and Uganda (n = 7)

Health outcomes indicators	Data Sources			Annual reporting			Disaggregation by Refugee status			Refugee population adjustment			Accuracy			Consistency		
	JOD	LBN	UG	JOD	LBN	UG	JOD	LBN	UG	JOD	LBN	UG	JOD	LBN	UG	JOD	LBN	UG
Neonatal mortality rate [SDG 3.2.2]	I	I & L	I & L	*✓*	✓^*^	*✓*	✕	*✓*^a^	✕	✕	*✓*	*✓*	*✓*	*✓*	*✓*	–	✕	✕
Infant mortality rate	I & L	I & L	I & L	*✓*	✓^*^	*✓*	✕	✕	✕	*✓* ^b^	✕	*✓*	*✓*	*✓*	*✓*	✕	✕	✕
Under 5 mortality rate – [SDG 3.2.1]	I	I & L	I & L	*✓*	✓^*^	*✓*	✕	✕	✕	✕	✕	*✓*	*✓*	*✓*	*✓*	–	✕	✕
Adolescent mortality rate	I	I	I	*✓*	*✓*	*✓*	✕	✕	✕	✕	✕	✕	*✓*	*✓*	*✓*	–	–	–
Maternal mortality ratio – [SDG 3.1.1]	I & L	I & L	I & L	✓^*^	✓^*^	*✓*	✕	*✓* ^a^	✕	*✓* ^b^	*✓*	*✓*	*✓*	*✓*	*✓*	✕	✕	*✓*
Adolescent birth rate – [SDG 3.7.2]	I	I & L	I	*✓*	✓^*^	*✓*	✕	✕	✕	✕	✕	✕	*✓*	*✓*	*✓*	–	✕	–
Total fertility rate	I & L	I	I & L	✓^*^	*✓*	✓^*^	✕	✕	✕	*✓* ^b^	✕	✕	*✓*	*✓*	*✓*	✕	–	*✓*

JOD, Jordan; LBN, Lebanon; UG, Uganda

*✓*, Yes; ✕, No/Not available; –, Not Applicable

I, International source only; L, Local source only

I & L, Both international and local sources available

^*^Reported annually from international sources but not reported annually from local sources/missing some years from local sources

^a^Indicator is disaggregated by Lebanese and non-Lebanese population regardless of refugee status

^b^Started accounting for refugee population in 2015

The lack of annual data reporting from local sources was observed for certain indicators. For instance, local data on maternal mortality ratio in Jordan was only available for the years 2012, 2014, 2015, and 2017, with no data available prior to the Syrian refugee crisis. Similarly, no local data was published for maternal mortality ratio during the years 2011–2016 in Lebanon, coinciding with the early years of the refugee crisis. In Uganda, fertility rate data was reported annually from international sources but only available for three years from local sources (2011, 2014, and 2016). This limited data across specific years poses challenges in identifying disparities in health outcomes between the host and refugee populations and prevents a comprehensive understanding of the impact of the refugee influx on population health outcomes. Additionally, discrepancies were observed between local and international sources when both were available for health outcomes indicators in Jordan and Lebanon, which presents challenges in ensuring data consistency. Specifically, discrepancies were observed for neonatal mortality rate, under-5 mortality rate per 1000 live births, and infant mortality rate per 1000 live births in Lebanon and Uganda, as well as for maternal mortality ratio per 100,000 live births in Jordan.

No local figures were found concerning adolescent mortality rate for the three countries; only international figures were available, mainly relying on estimates and projections without accounting for the refugee populations. Moreover, no local data on adolescent birth rate was found for either Jordan or Uganda, and no local data on fertility rate was found for Lebanon.

## Discussion

This study contributes to the limited literature on national HIS and refugee health, providing valuable insights into the strengths and weaknesses of current health information systems in the context of refugee crises. Our analysis revealed significant challenges in using routine data to analyze and review the health status of refugees in Jordan, Lebanon, and Uganda. Notably, we found that certain indicators lack data collection and reporting from local sources, requiring reliance on international sources that frequently rely on modelling approaches to generate indicators. We also identified fragmentation of sources that collect refugee-specific health data and discrepancies between local and international sources when both were available. Specifically, significant discrepancies were observed between local data (MOH, national HIS, department of statistics, etc.) and data from international sources (e.g., WHO, World Bank), including refugee-specific sources across the three countries (UNHCR). These discrepancies could be attributed to several factors, including variations in sample sizes used and the use of different indicator definitions. Discrepancies can also stem from different data collection methodologies. For example, local sources may use direct survey methods that provide immediate, context-specific insights, while international sources might employ standardized surveys or aggregate data collection techniques that aim for comparability across countries and populations. Such disparities highlight the need for standardization and harmonization of data collection and reporting practices across the different entities to ensure accurate and comparable health information on both refugee and host populations.

We observed outliers and sudden changes in certain indicators with no clear explanation; while this may be caused by errors in data collection, data entry, or data reporting, it is possible that revisions to indicators so as to include refugee populations also contribute to these sudden changes. There may be serious problems with the accuracy and consistency of health financing and economic indicators across the three countries, which could negatively affect the development of appropriate health financing policies and the development of suggestions on how to reallocate resources at the national level efficiently.

One of the critical limitations identified in our study is the lack of distinction between refugees and the host population in most national health data sources across Jordan, Lebanon, and Uganda. While some indicators include the refugee population within overall national figures, very few differentiate between refugees and the host community. This hinders our ability to directly compare health indicators and assess the specific health services available to refugees versus the host population. This limitation extends across various data categories. Our analysis found no segregation by refugee status for selected indicators on health service availability, health workforce, or health financing and economics in any of the three countries. Even among the limited indicators segregated by nationality (e.g., births attended by skilled personnel in Lebanon and neonatal/maternal mortality data), there are significant shortcomings. In Lebanon, for example, nationality data only differentiates between Lebanese and non-Lebanese, failing to provide details on specific refugee populations like Syrians. This lack of detail within the "non-Lebanese" category renders it unsuitable for representing the specific health outcomes of Syrian refugees in Lebanon. Additionally, the timeframe for this disaggregated data is restricted (2015–2018 for neonatal and maternal mortality and a single year for births attended by skilled personnel), limiting meaningful comparisons and analysis.

### Recent advancements in National health information systems in the three countries after the year 2019

Our analysis ended in 2019. Although there have been developments in the HS in Jordan and increased challenges in Lebanon due to the economic crisis, these are unlikely to have had major consequences for how effectively the HIS captures refugee populations. After the year 2019, additional efforts have been pursued in Jordan to widen the implementation of electronic healthcare records (EHRs) in hospitals across different regions of the country (40). However, many challenges have persisted such as the lack of clear guidelines for implementing EHRs, healthcare providers’ resistance to the new technology, inadequate financial resources, system configuration and technology infrastructure, inadequate training and technical support, and workflow and organizational structure [40, 45, 46]. On the other hand, Lebanon has recently experienced an economic and financial crisis that started in 2019, classified among the most severe globally since the mid-nineteenth century [74, 75]. As a result, all sectors have been negatively affected including the health sector; exacerbating the challenges that were already faced at the level of the national health information system [76].

In Uganda however, there have been developments related to refugees’ health data in the HMIS. Specifically, additional efforts have been pursued after 2019 to harmonize the Ugandan National Health Management Information System (UHMIS) with the UNHCR Refugee Health Information System (RHIS) which is a combined paper-based and electronic system used mainly in refugee hosting districts [21, 77, 78]. This is considered a major step for Uganda in advancing its refugee health data management; yet, it has been reported that the parallel use of UNHCR RHIS and UHMIS systems resulted in duplicate and inconsistent reporting of data, especially in high-volume facilities with heavy workloads, which affected the quality of data [21]. Additional challenges that have hindered the adoption and implementation of a single system platform at the level of the national health information system in Uganda include inadequate funding, limited infrastructure and technical capacity, insufficient human resources, and the lack of inclusiveness in capacity building plans [21, 48, 55].

### Implications for policy and practice

The study findings highlight the critical need for strengthened data collection and reporting systems for refugee health data in Jordan, Lebanon, and Uganda, especially given the protracted nature of the refugee situations in these three countries. Robust national HIS are essential for capturing data on vulnerable groups, including refugees, and understanding their health needs [1, 21]. Moreover, comprehensive national data on the impact of hosting refugees on health systems and financing is crucial for governments. This information allows them to present data-driven arguments to international partners, advocating for increased financial support to address the healthcare needs of their refugee populations [79, 80]. In light of the identified challenges and the importance of strong national HIS that integrate refugee health data, we propose a set of actionable recommendations for policymakers and practitioners in the three countries.

#### Establishment of a centralized authority for refugee health data management

Currently, data collection and reporting across the three countries involve multiple institutions, which is causing inconsistencies and inefficiencies. Therefore it is recommended that a national body or a central authority be designated to oversee data collection on refugee health and to standardize and validate data generated from different sources [33]. This approach, similar to advancements in national statistics departments of OECD countries can significantly improve data coordination, harmonization, and ultimately, data quality [81, 82]. Common indicators and consistent measurement approaches would enhance the monitoring of health status and healthcare outcomes, supporting evidence-based policymaking and targeted interventions [11, 14]. Furthermore, such a national body would facilitate stratified data analysis, allowing for the identification of at-risk refugee subgroups [14]. This disaggregated data would also enable comparisons of health outcomes between refugees and the host population [83]. The study identified a significant scarcity of data on crucial refugee health indicators, such as mental health workforce rates, support for health facilities by humanitarian organizations, contraceptive prevalence rates, impoverishing health expenditures, adolescent mortality and birth rates. A national body would be well-positioned to collect and report on this critical data, which plays a vital role in identifying refugee health needs and informing effective health system policies. This is particularly important for refugee populations, who are often vulnerable and marginalized.

#### Fostering transparency and inclusive data governance

Effectively integrating refugee health data into national health information systems requires transparency and collaboration across a broad range of stakeholders. This includes governments, international organizations, academic institutions, civil society groups, and other relevant actors [14, 84]. This collaborative effort should also include the active involvement of refugees themselves, along with their representatives, such as non-governmental organizations (NGOs) [12, 14]. This approach, often referred to as participatory data governance, empowers refugees to become key partners in data collection, analysis, and dissemination [85]. Their involvement helps ensure that public health initiatives are truly relevant to their needs. A participatory approach can overcome barriers like language and cultural differences, fostering trust and promoting equity within the data governance process [85]. Ultimately, this collaborative and transparent approach leads to more robust and meaningful data collection, analysis, and utilization of refugee health information.

#### Strengthening workforce capacity for refugee health data management

A critical challenge reported in the literature across the three countries was the lack of trained personnel skilled in managing health data, particularly refugee health data [49, 86, 87]. This skills gap hinders effective data collection, analysis, and utilization. To address this, there is a need to invest in training for healthcare workers at all levels. This capacity-building effort should equip them with the necessary skills to effectively manage health data including refugee health data [88]. A crucial component of the program should focus on building trust with refugee communities and fostering cultural competency in data collection practices. Culturally competent data collection ensures the gathered information accurately reflects the experiences and health needs of the refugee population [89]. By fostering trust and cultural sensitivity, healthcare workers can collect more accurate and comprehensive data. This, in turn, leads to improved decision-making, development of targeted interventions, and ultimately, better health outcomes for refugees [89].

#### Holistic integration of refugee health data with a focus on privacy

Integrating refugee health data into national health information systems is a crucial step, but it should be part of a broader strategy for addressing the unique health needs of refugees and their successful integration into host country health systems [90, 91]. This includes ensuring access to healthcare services, addressing social determinants of health, and promoting policies that support refugee integration into host communities. Strengthening the health information system to include refugee data ultimately benefits the entire population, including other vulnerable groups [90, 91]. It is also critical to acknowledge the potential risks associated with refugees revealing their status when seeking healthcare services. Robust safeguards must be implemented to prevent the misuse of refugee health data for non-health purposes and protect the privacy of refugee populations, ensuring that the integration of data does not harm their access to healthcare services or their legal status [85, 90, 91]. A compassionate asylum and healthcare system is essential, one that prioritizes the needs of the most disadvantaged, considering their unique social, economic, and legal situations [90, 91]. This requires a holistic approach that balances the need for data collection with robust privacy protections.

### Implications for research

In order to harness the full potential of available data sources and capture the diverse range of health outcomes within refugee settings, further research is warranted to explore the feasibility and effectiveness of integrating refugee registration data with other data sources, such as humanitarian survey data and facility-based data [81]. This integration could provide a more comprehensive understanding of refugee health and enable a more nuanced analysis of health outcomes and service utilization patterns.

One avenue for future investigation is the exploration of the compatibility and alignment of refugee registration data with data collected through humanitarian surveys. By combining these two sources of information, researchers can gain a more holistic view of the health needs and experiences of refugees, capturing not only demographic and administrative data but also valuable insights into health behaviors, access to healthcare, and health outcomes. This integration has the potential to enhance the accuracy and completeness of refugee health data and enable more informed decision-making in healthcare planning and resource allocation. Furthermore, collaboration between humanitarian agencies, national health authorities, and researchers is essential to establish data-sharing mechanisms, develop standardized protocols, and ensure ethical considerations in data integration processes.

However, it is important to acknowledge the methodological and practical challenges associated with data integration. Issues such as data compatibility, privacy concerns, data quality, and standardization of data collection methods need to be carefully addressed in future research endeavors.

## Conclusion

This is the first study to comprehensively examine the strengths and weaknesses of national health information systems in terms of integrating refugee health data and tracking the impact of the refugee crisis on the national health systems of three refugee-dense countries (Jordan, Lebanon, and Uganda).

In summary, this study identified several limitations in the national health information systems of Jordan, Lebanon, and Uganda regarding data collection, analysis, and reporting of refugee health data, as well as data use for decision-making. Addressing these limitations and strengthening health information systems can lead to better decision-making and improved health outcomes for all populations, including refugees.

These findings can inform the governments of the three countries regarding gaps in their health information systems and provide recommendations for improvement. The study findings, in terms of shortcomings and areas for improvement, can be applied to other countries hosting large number of refugees in the region and beyond.

### Supplementary Information

Additional file 1.

Additional file 2.

## Data Availability

Data is available upon reasonable request from the corresponding author.

## Abbreviations

CDC: Centers for Disease Prevention and Control
CHE: Current health expenditure
DHIS2: District Health Information System 2
DTP3: Diphtheria tetanus toxoid and pertussis
EHRs: Electronic healthcare records
GDP: Gross domestic product
GHO: Global Health Observatory
HIS: Health information systems
HMIS: Health Management Information System
MOH: Ministry of Health
NGOs: Non-governmental organizations
OECD: Organisation for Economic Co-operation and Development
PAHO: Pan American Health Organization
RHIS: Refugee Health Information System
UHMIS: Ugandan National Health Management Information System
UNHCR: United Nations High Commissioner for Refugees
UNICEF: United Nations International Children's Emergency Fund
WB: World Bank
WFP: World Food Programme
WHO: World Health Organization
