# Supplementary material for: Assessing the integration of refugee health data into national health information systems in Jordan, Lebanon, and Uganda
Source: Confl Health. 2024 Aug 5;18(Suppl 1):49. doi: 10.1186/s13031-024-00608-2 (PMC11299268; doi:10.1186/s13031-024-00608-2)
Supplement: Supplementary file 2 — Additional file 2. [file 13031_2024_608_MOESM2_ESM.docx]

**Appendix I- Selected indicators, definitions, strengths and weaknesses across the three countries**

| **Indicator** | | | **Definition** | **Comment/limitation of indicator** |
| --- | --- | --- | --- | --- |
| **Health Service Availability** | **1.** | **Number and distribution of health facilities per 10,000 population** | Total number of public and private health posts, health centers, district/rural hospitals, provincial hospitals, and regional/specialized/teaching and research hospitals per 10,000 population  The indicator does not include mobile service delivery points and non-formal services such as traditional healers. | **Jordan:** indicator not available **Lebanon:** indicator not available  **Uganda:** indicator not available |
|  | **2.** | **Number of inpatient beds per 10,000 population** | The number of hospital beds available per every 10 000 inhabitants in a population.  Hospital beds are used to indicate the availability of inpatient services. There is no global norm for the density of hospital beds in relation to total population. | **Jordan:** indicator available from local sources (MOH- HMIS) and from international sources (WB); data is reported annually; indicator not disaggregated by refugee status; the number of hospital beds reported by MOH started accounting for the Syrian refugee population; only one outlier was observed when data started accounting for refugee population thus data can be considered to be accurate; no discrepancies between the reported values from local sources and international sources thus data considered to be consistent **Lebanon:** indicator available from local sources (MOH- statistics department) and from international sources (WB); data is reported annually; indicator not disaggregated by refugee status; the number of hospital beds reported by MOH started accounting for Syrian refugees’ population in 2016 which is the last year reported; outliers were observed between the years 2012 and 2016 in Lebanon, for both local and international sources thus data cannot be considered accurate; there is great disparity between the local and international sources thus data cannot be considered to be consistent. **Uganda:** indicator available from local sources (MOH- HMIS) and from international sources (WB); data is reported annually from local sources but only available for two years from international sources (2009 and 2010); data not disaggregated by refugee status; reported data is free from outliers thus can be considerate to be accurate; in terms of consistency data is available from both local and international sources for the year 2010 only and there is disparity between the two values thus data cannot be considered to be consistent |
|  | **3.** | **Number and Percentage of health facilities supported by humanitarian organizations** | Indicator of support by health cluster partners beside MoH to the health system; in very disrupted health system can be a proxy for functional health facilities/services as non-supported health facilities have stopped functioning  **Numerator:** Number of health facilities in the same administrative or health area at a given point in time  **Denominator:** Number of health facilities supported by stakeholders other than MoH in a defined administrative or health area at a given point in time | **Jordan:** Indicator not available; only information available is related to UNRWA (Palestinian refugees)  **Lebanon:** Indicator not available **Uganda:** Indicator not available |
|  |  |  |  |  |
|  |  |  |  |  |
|  | **4.** | **Density of district/rural hospitals (per 100,000 population)** | Number of district/rural hospitals from the public and private sectors, per 100,000 population.  **Method of measurement:** Count of district/rural hospitals in the country, divided by the number of population. | **Jordan:** Indicator only available from international source (WHO-GHO); reported for two years only (2010 and 2013); indicator is not disaggregated by refugee status; data does not account for Syrian refugees population; reported data is free from outliers thus can be considerate to be accurate; consistency cannot be assessed since it is available from one source only.  **Lebanon:** Indicator only available from international source (WHO-GHO); reported for two years only (2010 and 2013); indicator is not disaggregated by refugee status; data does not account for Syrian refugees population; reported data is free from outliers thus can be considerate to be accurate; consistency cannot be assessed since it is available from one source only. **Uganda:** indicator available from both local and international sources; local sources data are reported annually while international data is only available for two years (2010 and 2013); indicator is not disaggregated by refugee status; data does not account for South Sudanese refugees population; reported data is free from outliers; however, there is great disparity between the local and international sources thus data cannot be considered to be consistent. |
|  | **5.** | **Density of primary health care facilities** | The number of public and private primary health care facilities available relative to the total population for the same geographical area.  **Numerator:** District and national databases provide the number of public primary health care facilities, often by type (such as, health center, PHC centers, health post, health houses, and dispensary). Special efforts, notably facility censuses, are often required to obtain the number of private facilities, especially if no registration system is enforced. A facility sample survey will not provide the data needed to compute service availability.  **Denominator:** Information collected directly from ministries of health through the baseline national health survey | **Jordan:** available from both local sources and international sources; data is reported annually; not disaggregated by refugee status; data does not account for Syrian refugees population; accuracy and consistency cannot be assessed since this indicator is reported as number from local sources and as density from international sources. **Lebanon:** available from both local sources and international sources; data is reported annually; not disaggregated by refugee status; data does not account for Syrian refugees population; accuracy and consistency cannot be assessed since this indicator is reported as number from local sources and as density from international sources. **Uganda:** available from local sources and not available from international sources; data is reported annually; not disaggregated by refugee status; data does not account for South Sudanese refugees population; reported data has outliers thus cannot be considerate to be accurate; consistency cannot be assessed since it is available from one source only. |
|  | **6.** | **Maternity bed density per 1,000 pregnant women** | Data on maternity beds can be used to calculate the density of maternal beds per 1,000 pregnant women per year. Public and private sectors are included. | **Jordan:** Indicator not available; only information available is related to UNRWA (Palestinian refugees)  **Lebanon:** Indicator not available **Uganda:** Indicator not available |
|  | **7.** | **Number of outpatient department visits per 10,000 population per year** | The number of outpatient visits to health facilities during one year relative to the total population of the same geographical area. Outpatient visit is defined as the contact with a health professional such as physicians (both generalists and specialists), nurse, midwife, dentists, etc., and is not admitted to any health care facility and does not occupy a hospital bed for any length of time. Health facilities include all public, private, non-governmental and community-based health facilities in which general health services are offered.  **Numerator:** Total number of outpatient department visits per year.  **Denominator:** Total population | **Jordan:** Indicator not available; only information available is related to UNRWA (Palestinian refugees)  **Lebanon:** Indicator not available **Uganda:** available from local sources for 2 years only (2013 and 2014); data is not reported annually; not disaggregated by refugee status; data does not account for South Sudanese refugees population; reported data is free from outliers thus can be considerate to be accurate; consistency cannot be assessed since it is available from one source only. |
| **Health Workforce** | **8.** | **Number of health workers per 10,000 population [SDG 3.c.1]** | The total number of physicians, nursing and midwifery personnel per 10 000 population The indicator is estimated using 2 indicator measurements: number of physicians, and, number of nursing and midwifery personnel. Measurement details of the 2 individual measurements are available respectively as density of physicians (per 1000 population) and density of nursing and midwifery personnel (per 1000 population). | **Jordan:** Indicator not available **Lebanon:** Available from Local sources for two years only not available from international sources; data is not reported annually; not disaggregated by refugee status; data does not account for Syrian refugees population; accuracy and consistency cannot be assessed since it is available from one source only and for two years only  **Uganda:** Indicator not available |
|  | **9.** | **Density of Physicians (per 10,000 population)** | The density of medical doctors is defined as the number of medical doctors, including generalists and specialist medical practitioners per 10,000 population in the given national and/or subnational area. | **Jordan:** available from both local sources and international sources; data is reported annually; not disaggregated by refugee status; data started accounting for Syrian refugees population in 2015; one outlier was observed when data started accounting for refugee population in 2015 thus data can be considered to be accurate; major discrepancies were observed between the reported values from local sources and international sources thus data cannot be considered to be consistent **Lebanon:** available from both local sources and international sources; data is reported annually; not disaggregated by refugee status; data does not account for Syrian refugees; reported data is free from outliers thus can be considerate to be accurate; there is disparity between the local and international sources thus data cannot be considered to be consistent **Uganda:** available from both local sources and international sources; data is reported annually; not disaggregated by refugee status; data does not account for South Sudanese refugees; reported data has outliers thus cannot be considerate to be accurate; there is disparity between the local and international sources thus data cannot be considered to be consistent |
|  | **10.** | **Density of Dentists (per 10,000)** | The density of dentists is defined as the number of dentists per 10,000 population in the given national and/or subnational area. | **Jordan:** available from both local sources and international sources; data is reported annually; not disaggregated by refugee status; data started accounting for Syrian refugees population in 2015; outliers observed thus data cannot be considered to be accurate; discrepancies were observed between the reported values from local sources and international sources thus data cannot be considered to be consistent **Lebanon:** available from both local sources and international sources; data is reported annually; not disaggregated by refugee status; data does not account for Syrian refugees; several outliers observed thus data cannot be considerate to be accurate; there is disparity between the local and international sources thus data cannot be considered to be consistent **Uganda:** data available from international sources only (WHO-GHO); data is not reported annually (only reported for two years (2016 and 2019); not disaggregated by refugee status; data does not account for refugees population; accuracy and consistency cannot be assessed since data is only available from one source and for two years. |
|  | **11.** | **Density of Pharmacists (per 10,000)** |  | **Jordan**: available from both local sources and international sources; data is reported annually; not disaggregated by refugee status; data started accounting for Syrian refugees population in 2015; outliers observed thus data cannot be considered to be accurate; discrepancies were observed between the reported values from local sources and international sources thus data cannot be considered to be consistent **Lebanon:** available from both local sources and international sources; data is reported annually; not disaggregated by refugee status; data does not account for Syrian refugees; several outliers observed thus data cannot be considerate to be accurate; there is disparity between the local and international sources thus data cannot be considered to be consistent **Uganda:** data available from international sources only (WHO-GHO); data is not reported annually (only reported for three years (2012, 2015 and 2018); not disaggregated by refugee status; data does not account for refugees population; accuracy and consistency cannot be assessed since data is only available from one source and for three years. |
|  | **12.** | **Density of nursing and midwifery personnel (per 10,000 population)** | The density of nursing and midwifery personnel is defined as the number of nursing and midwifery personnel per 10,000 population in the given national and/or subnational area. | **Jordan:** available from both local sources and international sources; data is reported annually; not disaggregated by refugee status; data started accounting for Syrian refugees population in 2015; no outliers observed thus data can be considered to be accurate; major discrepancies were observed between the reported values from local sources and international sources thus data cannot be considered to be consistent **Lebanon:** available from both local sources and international sources; data is reported annually; not disaggregated by refugee status; data does not account for Syrian refugees; reported data is free from outliers thus can be considerate to be accurate; there is great disparity between the local and international sources thus data cannot be considered to be consistent **Uganda:** available from both local sources and international sources; data is reported annually; not disaggregated by refugee status; data does not account for South Sudanese refugees; reported data is free from outliers thus can be considerate to be accurate; there is no major disparity between the local and international sources thus data can be considered to be consistent |
|  | **13.** | **Number of Medical and Pathology Laboratory Technicians** | Includes medical and pathology laboratory technicians in the given national and/or subnational area. Depending on the nature of the original data source may include practicing (active) only or all registered in the health occupation | **Jordan:** Indicator not available (it was only available from international source (WHO-GHO) for the year 2004 which out of the scope included in this study thus was not assessed)  **Lebanon:** Indicator not available **Uganda:** data available from international sources only (WHO-GHO); data is not reported annually (only reported for two years (2015 and 2018); not disaggregated by refugee status; data does not account for refugees population; accuracy and consistency cannot be assessed since data is only available from one source and for two years. |
|  | **14.** | **Psychiatrists working in mental health sector (per 100,000)** | Psychiatrists working in mental health (per 100,000 population), including professionals working in private and public mental health facilities as well as private practice. | **Jordan:** Only available from international sources(WHO-GHO); not reported annually (only available for one year 2016); not disaggregated by refugee status; data does not account for refugees population; accuracy and consistency cannot be assessed since data is only available from one source and for one year. **Lebanon:** Only available from international sources(WHO-GHO); not reported annually (only available for one year 2016); not disaggregated by refugee status; data does not account for refugees population; accuracy and consistency cannot be assessed since data is only available from one source and for one year. **Uganda:** Only available from international sources(WHO-GHO); not reported annually (only available for one year 2016); not disaggregated by refugee status; data does not account for refugees population; accuracy and consistency cannot be assessed since data is only available from one source and for one year. |
| **Health Service Coverage/Access** | **15.** | **Primary health care utilization threshold** | The threshold is a measure of health service use, calculated in terms of the average number of primary health care visits per year as a proportion of the population.  If the rate is lower than expected, it may indicate that the population does not have adequate access to health services. If the rate is high, it may suggest that the population is ‘overusing' health services. This may be due to the presence of a specific public health problem or because the population has been underestimated. | **Jordan:** Available from local sources only (MOH) but reported as number of PHC consultations and not as utilization threshold; number is reported annually, not disaggregated by refugee status; data account for refugees population since data is collected by each center regardless of nationality; accuracy and consistency cannot be assessed since data is reported as number and is only available from one source.  **Lebanon:** Available from local sources only (MOH-statistics department) but reported as number of PHC consultations and not as utilization threshold; number is reported annually, not disaggregated by refugee status; data account for refugees population since data is collected by each center regardless of nationality; accuracy and consistency cannot be assessed since data is reported as number and is only available from one source.  **Uganda:** Indicator not available |
|  | **16.** | **Coverage of DTP3** | The percentage of one-year-olds who have received three doses of the combined diphtheria, tetanus toxoid and pertussis (DTP3) vaccine in a given year.  **Numerator:** Number of children aged 12–23 months receiving three doses of the DTP vaccine. **Denominator:** Total number of children aged 12–23 months surveyed. | **Jordan:** available from both local sources and international sources; data is reported annually; not disaggregated by refugee status; data accounts for Syrian refugees population; outliers observed thus data cannot be considered to be accurate; no major discrepancies were observed between the reported values from local sources and international sources thus data can be considered to be consistent **Lebanon:** available from both local sources and international sources; data is reported annually; not disaggregated by refugee status; data accounts for Syrian refugees population; no outliers observed thus data can be considered to be accurate; no discrepancies were observed between the reported values from local sources and international sources thus data can be considered to be consistent **Uganda:** available from both local sources and international sources; data is reported annually; not disaggregated by refugee status; data accounts for refugees population; outliers observed thus data cannot be considered to be accurate; no major discrepancies were observed between the reported values from local sources and international sources thus data can be considered to be consistent |
|  | **17.** | **Contraceptive prevalence rate** | The percentage of women aged 15-49 years, married or in-union, who are currently using, or whose sexual partner is using, at least one method of contraception, regardless of the method used. | **Jordan:** Indicator available from international source only (WB); not reported annually (three years only: 2009, 2012 and 2018); not disaggregated by refugee status; data does not account for refugees population; accuracy and consistency cannot be assessed since data is only available from one source and for three years.  **Lebanon:** Indicator available from international source only (WB); not reported annually (one year only 2009); not disaggregated by refugee status; data does not account for refugees population; accuracy and consistency cannot be assessed since data is only available from one source and for three years.  **Uganda:** Indicator available from international source only (WB); not reported annually (missing years: 2009, 2010, 2012, 2013 and 2019); not disaggregated by refugee status; data does not account for refugees population; accuracy and consistency cannot be assessed since data is only available from one source and for three years |
|  | **18.** | **Antenatal care coverage** | The percentage of women aged 15-49 with a live birth in a given time period that received antenatal care four or more times. Due to data limitations, it is not possible to determine the type of provider for each visit.  **Numerator:** The number of women aged 15-49 with a live birth in a given time period that received antenatal care four or more times.  **Denominator:** Total number of women aged 15-49 with a live birth in the same period. | **Jordan:** available from international sources only (WHO), data not reported annually (only available for two years (2012 and 2017); not disaggregated by refugee status; data does not account for refugees population; accuracy and consistency cannot be assessed since data is only available from one source and for two years  **Lebanon:** Indicator not available (Available indicator in Lebanon has a different definition (number of pregnant women who visited PHC for more than one ANC visit) also the information available is for only PHC visits which doesn't represent the majority of the population)  **Uganda:** Indicator available from local source only (MOH HIS); reported annually; not disaggregated by refugee status; data accounts for refugees population; outliers observed thus data cannot be considered to be accurate; consistency cannot be assessed since data is only available from one source |
|  | **19.** | **Percentage of deliveries by caesarean section** | Percentage of births by caesarean section among all live births in a given time period. The percentage of births by caesarean section is an indicator of access to and use of emergency health care during childbirth. | **Jordan:** data available from international sources only (WHO-GHO)**;** data reported annually (latest reported data is 2017); not disaggregated by refugee status; data does not account for refugees population; data free from outliers thus can be considered to be accurate; consistency cannot be assessed since data is only available from one source  **Lebanon:** available from both local and international sources, data reported annually; not disaggregated by refugee status; data does not account for refugees population; data free from outliers thus can be considered to be accurate; no discrepancies observed between local and international sources thus data could be considered to be consistent.  **Uganda:** available from both local and international sources, data reported annually; not disaggregated by refugee status; data does not account for refugees population; outliers observed thus cannot be considered to be accurate; major discrepancies observed between local and international sources thus data could not be considered to be consistent. |
|  | **20.** | **Births attended by skilled health personnel [SDG 3.1.2]** | Proportion of births attended by skilled health personnel (generally doctors, nurses or midwives but can refer to other health professionals providing childbirth care) is the proportion of childbirths attended by professional health personnel. According to the current definition (1) these are competent maternal and newborn health (MNH) professionals educated, trained and regulated to national and international standards. They are competent to: (i) provide and promote evidence-based, human-rights based, quality, socio-culturally sensitive and dignified care to women and newborns; (ii) facilitate physiological processes during labour and delivery to ensure a clean and positive childbirth experience; and (iii) identify and manage or refer women and/or newborns with complications. | **Jordan:** available from international sources only (WHO-GHO); not reported annually (only available for two years 2012 and 2018); not disaggregated by refugee status; data does not account for refugees population; accuracy and consistency cannot be assessed  **Lebanon:** Available from international sources only (WHO and UNICEF); not reported annually (2009, 2011, 2016 and 2018); disaggregated by refugee status (for one year only 2018); the other values do not account for Syrian refugees population; reported data is free from outliers thus can be considered to be accurate; consistency cannot be assessed since it is reported from one source.  **Uganda:** available from both local and international sources, not reported annually (missing years 2012, 2013, 2014 and 2019), not disaggregated by refugee status, local data accounts for refugees population, outliers were not observed thus data can be considered to be accurate, major discrepancies between local and international data thus data cannot be considered consistent. |
|  | **21.** | **Access to a core set of relevant essential medicines [SDG 3.b.3]** | Proportion of health facilities that have a core set of relevant essential medicines available and affordable on a sustainable basis. The indicator is a multidimensional index reported as a proportion (%) of health facilities that have a defined core set of quality-assured medicines that are available and affordable relative to the total number of surveyed health facilities at national level. | **Jordan:** available from international sources only (WHO-EMRO); reported for one year only (2013); not disaggregated by refugee status, does not account for refugee population; accuracy and consistency cannot be assessed.  **Lebanon:** available from international sources only (WHO-EMRO); reported for one year only (2013); not disaggregated by refugee status, does not account for refugee population; accuracy and consistency cannot be assessed.  **Uganda:** available from local sources only; reported annually, not disaggregated by refugee status, one outlier observed thus cannot be considered to be accurate; consistency cannot be assessed since data is reported from one source only |
| **Health Financing and Economic Indicators** | **22.** | **Current Health Expenditure (CHE) as % Gross Domestic Product (GDP)** | Level of Current Health Expenditure expressed as a percentage of GDP. Current health expenditure as a share of GDP provides an indication on the level of resources channeled to health relative to other uses. It shows the importance of the health sector in the whole economy and indicates the societal priority which health is given measured in monetary terms. | **Jordan:** available from both local and international sources; data reported annually from international sources but missing years from local sources (no local data available before the year 2013 or after 2017, missing data for 2014); data not disaggregated by refugee status; data does not account for refugee population; no outliers observed thus can be considered to be accurate; discrepancies observed between local and international sources thus cannot be considered to be consistent.  **Lebanon:** available from both local and international sources; data reported annually from international sources but missing years from local sources (no local data available before the year 2012or after 2017, missing data for 2013 and 2014); data not disaggregated by refugee status; data does not account for refugee population; no outliers observed thus can be considered to be accurate; discrepancies observed between local and international sources thus cannot be considered to be consistent.  **Uganda:** available from both local and international sources; data reported annually from international sources but missing years from local sources (no local data available before the year 2011); data not disaggregated by refugee status; data does not account for refugee population; no outliers observed thus can be considered to be accurate; discrepancies observed between local and international sources thus cannot be considered to be consistent. |
|  | **23.** | **Current Health Expenditure (CHE) per Capita in US$** | Per capita current expenditures on health expressed in respective currency - US dollar. This indicator calculates the average expenditure on health per person. It contributes to understand the health expenditure relative to the population size facilitating international comparison. | **Jordan:** available from both local and international sources; data reported annually from international sources but missing years from local sources (no local data available before the year 2012 or after 2017, missing data for 2014); data not disaggregated by refugee status; data does not account for refugee population; no outliers observed thus can be considered to be accurate; discrepancies observed between local and international sources thus cannot be considered to be consistent.  **Lebanon:** available from both local and international sources; data reported annually from international sources but missing years from local sources (no local data available before the year 2012 or after 2017, missing data for 2013 and 2014); data not disaggregated by refugee status; data does not account for refugee population; no outliers observed thus can be considered to be accurate; discrepancies observed between local and international sources thus cannot be considered to be consistent.  **Uganda:** available from both local and international sources; data reported annually from international sources but missing years from local sources (no local data available before the year 2011); data not disaggregated by refugee status; data does not account for refugee population; no outliers observed thus can be considered to be accurate; discrepancies observed between local and international sources thus cannot be considered to be consistent. |
|  | **24.** | **Domestic Health Expenditure as % of Current Health Expenditure** | Share of current health expenditures funded from domestic sources. | **Jordan:** indicator not available **Lebanon:** indicator not available  **Uganda:** indicator not available |
|  | **25.** | **Domestic General Government Health Expenditure as % Current Health Expenditure** | Share of current health expenditures funded from general government sources, social health insurance. The share of Domestic General Government resources used to fund health expenditures of total current health expenditures indicates how much resources is the public sector devoting for health. Public sources include domestic revenue as internal transfers and grants, transfers, subsidies to voluntary health insurance beneficiaries, NPISH or enterprise financing schemes as well as social health insurance contributions. All these transfers and subsidies represent public sources for health and indicate the overall governments contribution to funding healthcare relative to other sources of funding from domestic private and external sources. | **Jordan:** available from both local and international sources; data reported annually from international sources but missing years from local sources (no local data available before the year 2012 or after 2017); data not disaggregated by refugee status; data does not account for refugee population; no outliers observed thus can be considered to be accurate; discrepancies observed between local and international sources thus cannot be considered to be consistent.  **Lebanon:** available from both local and international sources; data reported annually from international sources but missing years from local sources (no local data available before the year 2012 or after 2017); data not disaggregated by refugee status; data does not account for refugee population; no outliers observed thus can be considered to be accurate; discrepancies observed between local and international sources thus cannot be considered to be consistent.  **Uganda:** available from both local and international sources; data reported annually from international sources but missing years from local sources (no local data available before the year 2011); data not disaggregated by refugee status; data does not account for refugee population; no outliers observed thus can be considered to be accurate; discrepancies observed between local and international sources thus cannot be considered to be consistent. |
|  | **26.** | **Domestic General Government Health Expenditure per Capita in US$** | This refers to the average amount of money spent by the general government of a country or region on health-related activities and services per person, measured in US dollars (USD). | **Jordan:** available from international sources only (WB); data reported annually from international sources; data not disaggregated by refugee status; data does not account for refugee population; no outliers observed thus can be considered to be accurate; consistency cannot be assessed  **Lebanon:** available from international sources only (WB); data reported; data not disaggregated by refugee status; data does not account for refugee population; no outliers observed thus can be considered to be accurate; consistency cannot be assessed  **Uganda:** available from international sources only; data reported annually; data not disaggregated by refugee status; data does not account for refugee population; no outliers observed thus can be considered to be accurate; consistency cannot be assessed |
|  | **27.** | **Domestic Private Health Expenditure as % Current Health Expenditure** | Share of current health expenditures funded from private sources. The share of domestic private expenditures on health of the total current health expenditures indicates how much is funded domestically by the private sector. Private sector funds stem from households, corporations and non-profit organizations. Such expenditures can be either prepaid to voluntary health insurance or paid directly to healthcare providers. This indicator describes the role of the private sector in funding healthcare relative to public or external sources. | **Jordan:** available from both local and international sources; data reported annually from international sources but missing years from local sources (no local data available before the year 2012 or after 2017, missing data for 2014); data not disaggregated by refugee status; data does not account for refugee population; no outliers observed thus can be considered to be accurate; discrepancies observed between local and international sources thus cannot be considered to be consistent.  **Lebanon:** available from both local and international sources; data reported annually from international sources but missing years from local sources (no local data available before the year 2012 or after 2017, missing data for 2013 and 2014); data not disaggregated by refugee status; data does not account for refugee population; no outliers observed thus can be considered to be accurate; discrepancies observed between local and international sources thus cannot be considered to be consistent.  **Uganda:** available from both local and international sources; data reported annually from international sources but missing years from local sources (no local data available before the year 2011); data not disaggregated by refugee status; data does not account for refugee population; no outliers observed thus can be considered to be accurate; discrepancies observed between local and international sources thus cannot be considered to be consistent. |
|  | **28.** | **Domestic Private Health Expenditure per Capita in US$** | Current private expenditures on health per capita expressed in current US dollars. Domestic private sources include funds from households, corporations and non-profit organizations. Such expenditures can be either prepaid to voluntary health insurance or paid directly to healthcare providers. | **Jordan:** available from international sources only (WB); data reported annually from international sources; data not disaggregated by refugee status; data does not account for refugee population; no outliers observed thus can be considered to be accurate; consistency cannot be assessed  **Lebanon:** available from international sources only (WB); data reported; data not disaggregated by refugee status; data does not account for refugee population; no outliers observed thus can be considered to be accurate; consistency cannot be assessed  **Uganda:** available from international sources only; data reported annually; data not disaggregated by refugee status; data does not account for refugee population; no outliers observed thus can be considered to be accurate; consistency cannot be assessed |
|  | **29.** | **Health Expenditure from External Sources as % of Current Health Expenditure** | Share of current health expenditures funded from external sources. The share of external sources spent on health as percentage of total current health expenditures indicates how much is the health system dependent on external funding sources relative to domestic sources. External sources compose of direct foreign transfers and foreign transfers distributed by government encompassing all financial inflows into the national health system from outside the country. | **Jordan:** available from both local and international sources; data reported annually from international sources but missing years from local sources (no local data available before the year 2012 or after 2017, missing data for 2014); data not disaggregated by refugee status; data does not account for refugee population; no outliers observed thus can be considered to be accurate; discrepancies observed between local and international sources thus cannot be considered to be consistent.  **Lebanon:** available from both local and international sources; data reported annually from international sources but missing years from local sources (no local data available before the year 2012 or after 2017, missing data for 2013 and 2014); data not disaggregated by refugee status; data does not account for refugee population; no outliers observed thus can be considered to be accurate; discrepancies observed between local and international sources thus cannot be considered to be consistent.  **Uganda:** available from both local and international sources; data reported annually from international sources but missing years from local sources (no local data available before the year 2011); data not disaggregated by refugee status; data does not account for refugee population; no outliers observed thus can be considered to be accurate; discrepancies observed between local and international sources thus cannot be considered to be consistent. |
|  | **30.** | **Health Expenditure from External Sources per Capita in US$** | Per capita current external expenditures on health expressed in respective currency – USD. This indicator calculates the average external sources spent on health per capita in USD currency. External sources compose of direct foreign transfers and foreign transfers distributed by government encompassing all financial inflows into the national health system from outside the country. This indicator describes the size of the external sources for health expenditures in relation to the population size facilitating international comparison. | **Jordan:** indicator not available **Lebanon:** indicator not available  **Uganda:** indicator not available |
|  | **31.** | **Out of pocket Expenditures as % Current Health Expenditure** | Share of out of pocket payments of total current health expenditures. This indicator estimates how much are households in each country spending on health directly out of pocket. It estimates the share of out of pocket payment of total current health expenditures. | **Jordan:** available from both local and international sources; data reported annually from international sources but missing years from local sources (no local data available before the year 2012 or after 2017, missing data for 2014); data not disaggregated by refugee status; data does not account for refugee population; no outliers observed thus can be considered to be accurate; discrepancies observed between local and international sources thus cannot be considered to be consistent.  **Lebanon:** available from both local and international sources; data reported annually from international sources but missing years from local sources (no local data available before the year 2012 or after 2017, missing data for 2013 and 2014); data not disaggregated by refugee status; data does not account for refugee population; no outliers observed thus can be considered to be accurate; discrepancies observed between local and international sources thus cannot be considered to be consistent.  **Uganda:** available from both local and international sources; data reported annually from international sources but missing years from local sources (no local data available before the year 2011); data not disaggregated by refugee status; data does not account for refugee population; no outliers observed thus can be considered to be accurate; discrepancies observed between local and international sources thus cannot be considered to be consistent. |
|  | **32.** | **Out-of-Pocket Expenditure per Capita in US$** | This indicator estimates the average health expenditure through out-of-pocket payments per capita in USD. It indicates how much every person pays out of pocket on average in USD at the point of use. High out of pocket payment are associated with catastrophic and impoverishing household spending. Out of pocket payment are not pooled and there is no sharing of risk among wider group of people other than the household. This indicator describes the OOP expenditure in relation to the population size in USD facilitating international comparison. | **Jordan:** indicator not available **Lebanon:** indicator not available  **Uganda:** indicator not available |
|  | **33.** | **Gross Domestic Product per Capita in US$** | Gross Domestic Product (GDP) per capita is a core indicator of economic performance and commonly used as a broad measure of average living standards or economic wellbeing; despite some recognized shortcomings. | **Jordan:** available from both local and international sources; data reported annually from international sources but missing years from local sources (no local data available before the year 2012 or after 2017, missing data for 2014); data not disaggregated by refugee status; data does not account for refugee population; no outliers observed thus can be considered to be accurate; discrepancies observed between local and international sources thus cannot be considered to be consistent.  **Lebanon:** available from both local and international sources; data reported annually from international sources but missing years from local sources (no local data available before the year 2012 or after 2017, missing data for 2013 and 2014); data not disaggregated by refugee status; data does not account for refugee population; no outliers observed thus can be considered to be accurate; discrepancies observed between local and international sources thus cannot be considered to be consistent.  **Uganda:** available from both local and international sources; data reported annually from international sources but missing years from local sources (no local data available before the year 2011); data not disaggregated by refugee status; data does not account for refugee population; no outliers observed thus can be considered to be accurate; discrepancies observed between local and international sources thus cannot be considered to be consistent. |
|  | **34.** | **Primary Health Care (PHC) Expenditure per Capita in US**$ | Primary Health Care (PHC) Expenditure per Capita in US$ refers to the average amount of money spent on primary health care services per person in a particular country or region, measured in US dollars (USD). Primary health care encompasses a range of essential health services, including promotive, preventive, curative, and rehabilitative services that are typically provided by a primary care provider, such as general practitioners, family physicians, or community health centers. | **Jordan:** indicator not available **Lebanon:** indicator not available  **Uganda:** indicator not available |
|  | **35.** | **Primary Health Care (PHC) Expenditure as % Current Health Expenditure (CHE)** | Primary Health Care (PHC) Expenditure as % Current Health Expenditure (CHE)" refers to the proportion or percentage of total current health expenditure that is allocated specifically to primary health care services in a given country or region. | **Jordan:** indicator not available **Lebanon:** indicator not available  **Uganda:** indicator not available |
|  | **36.** | **Domestic General Government Expenditure on primary health care as % Domestic General Government Health Expenditure** | This refers to the proportion or percentage of domestic general government health expenditure that is allocated specifically to primary health care services. | **Jordan:** indicator not available **Lebanon:** indicator not available  **Uganda:** indicator not available |
|  | **37.** | **Proportion of the population with impoverishing health expenditure** | Proportion of the population where a household’s total consumption expenditure or income including household expenditure on health is greater than the poverty line but the household’s total consumption expenditure or income excluding household expenditure on health is below the poverty line.  **Numerator:** Total number of people whose household’s total consumption expenditure or income including household expenditure on health is greater than the poverty line but the household’s total consumption expenditure or income excluding household expenditure on health is below the poverty line.  **Denominator:** Total number of people. | **Jordan:** indicator not available **Lebanon:** indicator not available  **Uganda:** indicator not available |
|  | **38.** | **Proportion of the population with large household expenditure on health as a share of total household consumption or income [SDG 3.8.2]** | The proportion of the population with household expenditure on health exceeding 10% of total household expenditure or income.  **Associated terms:** Catastrophic health spending, Financial hardship, Financial protection, Out-of-pocket health expenditure | **Jordan:** indicator not available **Lebanon:** indicator not available  **Uganda:** indicator not available |
| **Health Outcomes for Hosts and Refugees** | **39.** | **Neonatal mortality rate [SDG 3.2.2]** | Number of deaths during the first 28 completed days of life per 1000 live births in a given year or another period. Neonatal deaths (deaths among live births during the first 28 completed days of life) may be subdivided into early neonatal deaths, occurring during the first 7 days of life, and late neonatal deaths, occurring after the 7th day but before the 28th completed day of life. | **Jordan:** available from international sources only; data is reported annually; not disaggregated by refugee status, does not account for refugee status; no outliers thus can be considered to be accurate; consistency cannot be assessed  **Lebanon:** available from both local and international sources (UNICEF); data reported annually however no local data before the year 2014; data is disaggregated between Lebanese and non-Lebanese population regardless of refugee status, local data accounts for refugee population, no outliers observed thus data can be considered to be accurate; discrepancies observed between local and international sources thus cannot be considered to be consistent  **Uganda:** available from local and international sources; reported annually; not disaggregated by refugee status; local data accounts for refugees; no outliers observed thus can be considered to be accurate; minor discrepancies between local and international sources thus cannot be considered to be consistent |
|  | **40.** | **Infant mortality rate** | Infant mortality rate is the probability of a child born in a specific year or period dying before reaching the age of one, if subject to age-specific mortality rates of that period. Infant mortality rate is strictly speaking not a rate (i.e. the number of deaths divided by the number of population at risk during a certain period of time) but a probability of death derived from a life table and expressed as rate per 1000 live births. | **Jordan:** available from both local and international sources; data is reported annually; not disaggregated by refugee status; local data started accounting for refugees population starting 2015; no outliers observed thus data can be considered to be accurate; discrepancies observed between local and international sources thus cannot be considered consistent.  **Lebanon:** available from both local and international sources; reported annually from international sources but missing years for local sources; not disaggregated by refugee status; data does not account for refugee population; no outliers observed thus data can be considered to be accurate; discrepancies observed between local and international sources thus cannot be considered to be consistent **Uganda:** available from local and international sources; reported annually; not disaggregated by refugee status; local data accounts for refugees; no outliers observed thus can be considered to be accurate; minor discrepancies between local and international sources thus cannot be considered to be consistent |
|  | **41.** | **Under 5 mortality rate – [SDG 3.2.1]** | The probability of a child born in a specific year or period dying before reaching the age of five, if subject to age-specific mortality rates of that period. Under-five mortality rate as defined here is strictly speaking not a rate (i.e. the number of deaths divided by the number of population at risk during a certain period of time) but a probability of death derived from a life table and expressed as rate per 1000 live births. | **Jordan:** available from international sources only; data is reported annually; not disaggregated by refugee status, does not account for refugee status; no outliers thus can be considered to be accurate; consistency cannot be assessed  **Lebanon:** available from both local and international sources; reported annually from international sources but missing few years for local sources; not disaggregated by refugee status; data does not account for refugee population; no outliers observed thus data can be considered to be accurate; discrepancies observed between local and international sources thus cannot be considered to be consistent  **Uganda:** available from local and international sources; reported annually; not disaggregated by refugee status; local data accounts for refugees population; no outliers observed thus can be considered to be accurate; minor discrepancies between local and international sources thus cannot be considered to be consistent |
|  | **42.** | **Adolescent mortality rate** | Number of deaths among adolescents (10-19 years old) per 100 000 adolescent population. | **Jordan:** Indicator available from international source only (WB); reported annually; not disaggregated by refugee status; data does not account for refugees population; no outliers observed thus data can be considered to be accurate; consistency cannot be assessed since data is only available from one source  **Lebanon:** Indicator available from international source only (WB); reported annually; not disaggregated by refugee status; data does not account for refugees population; no outliers observed thus data can be considered to be accurate; consistency cannot be assessed since data is only available from one source  **Uganda:** Indicator available from international source only (WB); reported annually; not disaggregated by refugee status; data does not account for refugees population; no outliers observed thus data can be considered to be accurate; consistency cannot be assessed since data is only available from one source |
|  | **43.** | **Maternal mortality ratio – [SDG 3.1.1]** | The maternal mortality ratio (MMR) is defined as the number of maternal deaths during a given time period per 100,000 live births during the same time period. It depicts the risk of maternal death relative to the number of live births and essentially captures the risk of death in a single pregnancy or a single live birth. | **Jordan:** available from both local and international sources; data is reported annually however several missing years from local sources; not disaggregated by refugee status; local data started accounting for refugees population starting 2015; no outliers observed thus data can be considered to be accurate; discrepancies observed between local and international sources thus cannot be considered consistent.  **Lebanon:** available from both local and international sources (UNICEF); data reported annually however no local data before the year 2011; data is disaggregated between Lebanese and non-Lebanese population regardless of refugee status, local data accounts for refugee population, no outliers observed thus data can be considered to be accurate; discrepancies observed between local and international sources thus cannot be considered to be consistent  **Uganda:** available from both local and international sources; data is reported annually; not disaggregated by refugee status; local data accounts for refugees population; no outliers observed thus data can be considered to be accurate; no discrepancies observed between local and international sources thus can be considered consistent. |
|  | **44.** | **Adolescent birth rate – [SDG 3.7.2]** | This indicator is defined as the annual number of births to women aged 15-19 years per 1000 women in that age group. It is also referred to as the age-specific fertility rate for women aged 15-19. | **Jordan:** data available from international source only (WB); data is reported annually; data not disaggregated by refugee status; data does not account for refugee population; no outliers observed thus data can be considered to be accurate; consistency cannot be assessed since data is available from one source only.  **Lebanon:** data available from local and international sources, data reported annually for international sources however only reported for two years from local sources; data not disaggregated by refugee status; data does not account for refugees; no outliers observed thus can be considered to be accurate; discrepancies between local and international sources thus data cannot be considered to be consistent.  **Uganda:** data available from international source only (WB); data is reported annually; data not disaggregated by refugee status; data does not account for refugee population; no outliers observed thus data can be considered to be accurate; consistency cannot be assessed since data is available from one source only. |
|  | **45.** | **Total fertility rate** | The average number of children a hypothetical cohort of women would have at the end of their reproductive period if they were subject during their whole lives to the fertility rates of a given period and if they were not subject to mortality. It is expressed as children per woman. | **Jordan:** available from local and international sources; data reported annually but missing two years from local sources (2011 and 2015); not disaggregated by refugee status; local data started accounting for refugee population starting 2015; no outliers observed thus data can be considered to be accurate; discrepancies observed between local and international sources thus data cannot be considered to be consistent.  **Lebanon:** available from international sources only (WB); data reported annually; data not disaggregated by refugee status; data does not account for refugee status; no outliers observed thus data can be considered to be accurate; consistency cannot be assessed since data is reported from one source.  **Uganda:** available from international and local sources; data reported annually from international sources but only for three years from local sources; not disaggregated by refugee status; does not account for refugee population; no outliers observed thus data can be considered to be accurate; no major discrepancies observed between local and international sources thus data can be considered to be consistent. |
